# Coordination modulation of iridium single-atom catalyst maximizing water oxidation activity

**DOI:** 10.1038/s41467-021-27664-z

**Published:** 2022-01-10

**Authors:** Zhanwu Lei, Wenbin Cai, Yifei Rao, Kuan Wang, Yuyuan Jiang, Yang Liu, Xu Jin, Jianming Li, Zhengxing Lv, Shuhong Jiao, Wenhua Zhang, Pengfei Yan, Shuo Zhang, Ruiguo Cao

**Affiliations:** 1 Hefei National Laboratory for Physical Sciences at the Microscale, CAS Key Laboratory of Materials for Energy Conversion, Department of Materials Science and Engineering, University of Science and Technology of China, Hefei, Anhui 230026 China; 2 Beijing Key Laboratory of Microstructure and Properties of Solids, Beijing University of Technology, Beijing, 100124 China; 3 Research Center of New Energy, Research Institute of Petroleum Exploration and Development (RIPED), PetroChina, Beijing, 100083 China; 4 Shanghai Synchrotron Radiation Facility, Shanghai Institute of Applied Physics, Chinese Academy of Sciences, Shanghai, 201204 China

**Keywords:** Catalyst synthesis, Electrocatalysis, Synthesis and processing

## Abstract

Single-atom catalysts (SACs) have attracted tremendous research interests in various energy-related fields because of their high activity, selectivity and 100% atom utilization. However, it is still a challenge to enhance the intrinsic and specific activity of SACs. Herein, we present an approach to fabricate a high surface distribution density of iridium (Ir) SAC on nickel-iron sulfide nanosheet arrays substrate (Ir_1_/NFS), which delivers a high water oxidation activity. The Ir_1_/NFS catalyst offers a low overpotential of ~170 mV at a current density of 10 mA cm^−2^ and a high turnover frequency of 9.85 s^−1^ at an overpotential of 300 mV in 1.0 M KOH solution. At the same time, the Ir_1_/NFS catalyst exhibits a high stability performance, reaching a lifespan up to 350 hours at a current density of 100 mA cm^−2^. First-principles calculations reveal that the electronic structures of Ir atoms are significantly regulated by the sulfide substrate, endowing an energetically favorable reaction pathway. This work represents a promising strategy to fabricate high surface distribution density single-atom catalysts with high activity and durability for electrochemical water splitting.

## Abstract (web summary)

Single-atom catalysts represent a promising materials class for efficient catalytic transformations. Here, authors prepare a high surface distribution density of iridium atoms on nickel-iron sulfide nanosheet arrays to deliver high water oxidation activity and stability.

## Introduction

Electrochemical water splitting is considered as a sustainable option for large-scale hydrogen production by using renewable energy, such as solar, wind and hydropower^1–3^. However, the sluggish kinetics of water electrolysis results in high overpotentials for both hydrogen evolution reaction (HER) and oxygen evolution reaction (OER)^4,5^, thus significantly sacrificing the energy efficiency and increasing the hydrogen production cost^6,7^. Especially, the OER reaction involves a four-electron process which suffers from ten times higher overpotentials than that of HER, making it a bottleneck process in the overall water electrolysis system^4,8^. The commercial electrocatalysts used for water oxidaton in alkaline electrolyzer are Ni-based catalysts^9^. However, their catalytic performance remains far from satisfactory for the cost competiveness of hydrogen production. Thus, tremendous efforts have been made to improve the OER performance^10^, thus achieving high-efficiency electrolysis for cost-effective hydrogen energy in alkaline electrolyzer. So far, the catalysts for OER need to utilize precious metals, such as iridium (Ir) and ruthenium (Ru), to suppress the overpential during the water electrolysis process^11^. The utilization of precious metal catalysts significantly impairs the cost competitiveness of hydrogen production via electrochemical water splitting in comparison with other conventional hydrogen production technologies from the steam reforming process^12^, thus hindering the wide deployment of the electrolytic hydrogen production technology in the future^13–15^.

Single-atom catalysts (SACs) provide a promising option to significantly reduce the utilization amount of precious metal catalysts by maximizing the atom-efficiency of precious metal elements in catalyst materials^16–21^. Recently, tremendous efforts have been devoted to developing efficient SACs for suppressing the OER overpotentials and improving the reaction kinetics for electrochemical water splitting^22–25^. It has been proved that the utilization of SACs could significantly improve the intrinsic activity of precious metal elements for the OER process and result in high mass activity^24,26,27^. The interaction between individual precious metal atoms and support substrate plays essential roles in modulating the microenvironment of active sites and consequently improves their activity and durability^28–30^. Various substrate materials have been attempted to maximize the mass activity of previous metals^31–35^. Despite these endeavors in developing suitable substrates for OER catalysts^17,36,37^, the fundamental understanding of the interaction between the substrate and the supported isolated atoms is still illusive^38–40^. Another major challenge in the fundamental science of SACs is to enhance the surface distribution density of active sites on the outer surface of substrates, rather than embedded inside the bulk materials^28,38^.

Herein, we report a facile and scalable strategy to fabricate a low mass loading but high surface distribution density Ir SAC by anchoring on the Ni_(3-x)_Fe_x_S_2_ (Ir_1_/NFS) nanosheet arrays via a two-step electrochemical method. The separation of substrate construction and Ir SAC deposition makes the distribution of Ir atoms only on the surface of the substrate, rather than inside the electrode, therefore maximizing the utilization of precious metals. As a result, the Ir_1_/NFS exhibits a high OER catalytic performance, which delivers an ultra-low overpotential of ~170 mV at a current density of 10 mA cm^−2^ with an ultra-high turnover frequency (TOF) of 9.85 s^−1^ at an overpotential of 300 mV. Density functional theory (DFT) calculations predict that the suitable chemical environment of Ir single-atom on the surface of NFS by Ir-S-M bond (M stands for Ni or Fe) efficiently reduces the kinetic energy barrier to form *OOH group from *O group, and thus accelerates the OER process.

## Results

### Catalysts fabrication and characterization

**Fig. 1:**
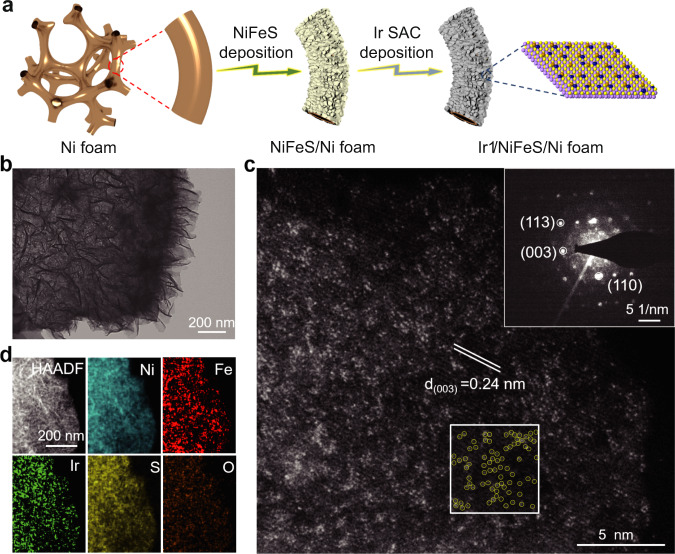
Electron microscope analysis of the Ir_1_/NFS. **a** Schematic illustration of the deposition to form the Ir_1_/NFS with two-step method. **b** TEM image of as-prepared the Ir_1_/NFS. Scale bar, 200 nm. **c** HAADF-STEM image of the Ir_1_/NFS (inset: SAED pattern) and the high density bright dots highlighted by yellow circles in an area of 5 nm × 5 nm. Scale bar, 5 nm. **d** HAADF image and the corresponding EDX elemental mappings of Ni, Fe, Ir, S, and O. Scale bar, 200 nm.

The fabrication process of Ir_1_/NFS on a Ni foam electrode is illustrated in Fig. 1a. In order to separate the substrate construction and Ir SAC deposition processes, the nickel-iron sulfide nanosheets were firstly deposited on a Ni foam electrode by sweeping the potentials between 0.2 V and −1.2 V versus Hg/HgO reference electrode in a solution containing thiourea (TU) and nickel-iron precursors. Subsequently, the iridium precursor was added into the electrolyte and the electrode was swept between 0.3 V and −0.3 V versus Hg/HgO reference electrode for Ir single-atoms deposition (see details in experimental methods). As a result, the Ir atoms were deposited on the surface of NFS substrate, rather than inside the electrode, therefore maximizing the utilization of Ir atoms. Notably, thiourea plays an essential role in forming the sulfide substrate during the first deposition step and stabilizing the Ir precursor in the electrolyte during the second deposition step. Raman spectra shows a negative shift (~10 cm^−1^) for the peak at 489 cm^−1^ with increasing thiourea concentration (Supplementary Fig. 1), indicating that Ir ions form the complex of Ir(TU)_x_ in the electrolyte^41^. The formation of complex ions prevents the formation of Ir clusters and nanoparticles during the deposition process. When without adding thiourea in the electrolyte, the Ir SAC on nickel-iron hydroxide substrate (Ir_1_/NFH) was formed after the same deposition procedure.

The scanning electron microscopy (SEM) characterization results reveal that the Ir_1_/NFS catalyst displays a highly porous surface morphology with interconnected wrinkles (Supplementary Fig. 2), which is helpful to expose high surface area and render more active sites. The corresponding transmission electron microscope (TEM) image further confirms that the Ir_1_/NFS nanosheets are composed of a fluffy ultrathin layered structure (Fig. 1b). The high-angle annular dark field-scanning transmission electron microscopy (HAADF-STEM) image reveals that the high-density isolated Ir atoms are homogeneously dispersed on the surface of NFS, without showing any aggregated clusters and nanoparticles (Fig. 1c, and Supplementary Fig. 3). The intensity line profile confirms that the Ir atom is located on the surface of NFS (Supplementary Fig. 4)^31^. Significantly, as shown in the framed area of Fig. 1c, the surface distribution density of Ir_1_ atoms reaches up to ~3 atoms per square nanometer, which is higher than most of ever-reported SACs in literatures^16,17,23^. As shown in the inset of Fig. 1c, the selected area electron diffraction (SAED) pattern presents the diffraction spots for (110), (113), and (003) planes from a hexagonal structure of NFS nanosheets. The elemental mapping results based on the energy-dispersive X-ray (EDX) further prove that the Ir element is uniformly distributed on the NFS nanosheets in a large scale area without showing any aggregation phenomena (Fig. 1d). Notably, the mass content of Ir in Ir_1_/NFS is determined to be ~0.6 wt% by the inductively coupled plasma mass spectrometry (ICP-MS) measurement.

For comparison, the morphology and structure of Ir_1_/NFH were also characterized by using SEM and TEM. The electrode with Ir_1_/NFH also shows porous surface morphology similar to the electrode with Ir_1_/NFS (Supplementary Fig. 5). However, the Ir_1_/NFH catalyst possesses thicker nanosheets in comparison to the Ir_1_/NFS catalyst (Supplementary Fig. 6). The HAADF-STEM images reveal that the isolated Ir atoms are distributed on the NFH nanosheets, showing less aggregated Ir clusters (Supplementary Figs. 7 and 8). Furthermore, the corresponding EDX elemental mappings also show a homogeneous Ir distribution in the Ir_1_/NFH catalyst (Supplementary Fig. 9). Notably, the Ir mass loadings in Ir_1_/NFH is ~0.54 wt% by ICP-MS measurement, which is very close to the Ir loading in Ir_1_/NFS. Based on these characterization results, it indicates that the sulfide substrate is more favorable for forming high surface distribution density Ir single-atom in comparison with the hydroxide substrate. In order to prove this hypothesis, various precious metal SACs on NFS were fabricated following the same synthesis procedure, including Ru_1_/NFS, Au_1_/NFS, and Pt_1_/NFS (Supplementary Figs. 10–12). The isolated Pt_1_, Au_1_, and Ru_1_ can be homogeneously dispersed on the surface of NFS, without showing any aggregated clusters and nanoparticles (Supplementary Figs. 13–15).

**Fig. 2:**
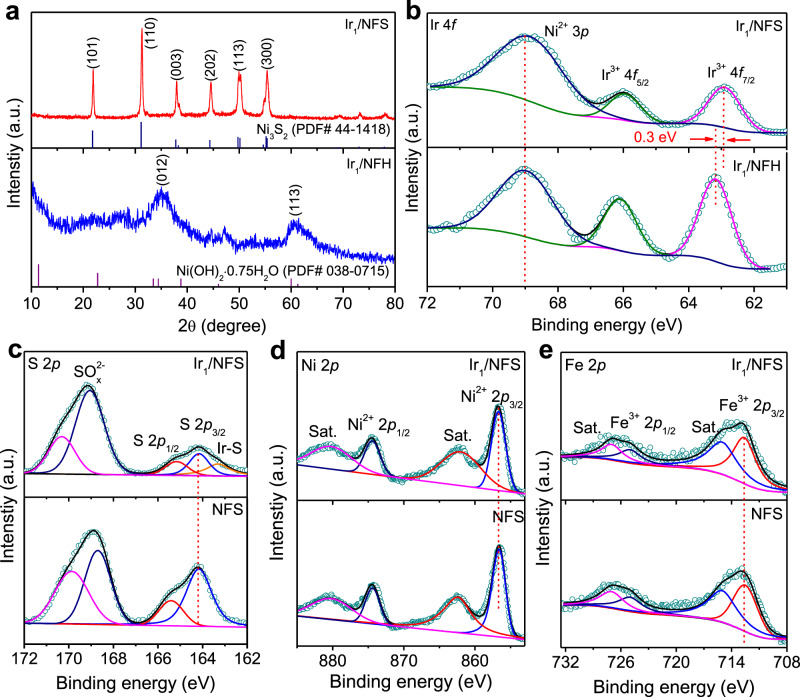
Physicochemical property characterization. **a** XRD pattern of the Ir_1_/NFS and Ir_1_/NFH. **b** XPS spectra of Ir 4*f* region with fitting curves for Ir_1_/NFS and Ir_1_/NFH. **c**–**e** XPS spectra of (**c**) S 2*p*, (**d**) Ni 2*p*, and (**e**) Fe 2*p* regions with fitting curves for Ir_1_/NFS and NFS.

The X-ray diffraction (XRD) patterns reveal that both Ir_1_/NFS and NFS possess the same crystalline structure that can be assigned to a crystal phase (PDF# 44-1418) of Ni_3_S_2_ (Fig. 2a and Supplementary Fig. 16), indicating that the electrochemical process for Ir SAC deposition does not change the structure of NFS substrate. In contrast, the XRD patterns of both Ir_1_/NFH and NFH show less crystalline structure with two broad peaks at 35° and 62°, which can be assigned to a crystalline structure of Ni(OH)_2_·0.75·H_2_O (PDF# 038-0715). X-ray photoelectron spectroscopy (XPS) was conducted to investigate the valence states of different elements in the catalysts. The core level Ir 4 *f* spectra of Ir_1_/NFS shows two peaks at 66.0 eV (Ir 4*f*_5/2_) and 62.9 eV (Ir 4*f*_7/2_), respectively. For comparison, the corresponding binding energy of Ir for Ir_1_/NFS is slightly lower than that for Ir_1_/NFH (63.2 eV for Ir 4 *f*_7/2_), confirming the weaker chemical interaction in Ir_1_/NFS than that in Ir_1_/NFH (Fig. 2b). To further investigate the valence state of Ir, the XPS measurements for IrCl_3_·xH_2_O and IrO_2_ were performed as references. It is observed that the valence state of Ir single-atoms in Ir_1_/NFS is slightly below the Ir (III) species in IrCl_3_·xH_2_O, and is much lower than the Ir (IV) species in IrO_2_ (Supplementary Figs. 17 and 18). XPS depth profiling spectra of Ir^3+^ 4 *f* show that the peak intensity of Ir^3+^ 4*f* decreases along with the increase of sputtering time while the peak intensity of Ni^2+^ 3*p* has no obvious change, which indicates that the Ir SACs are located on the surface of NFS nanosheets (Supplementary Fig. 19). In addition, the high-resolution XPS S 2*p* spectrum implies the formation of Ir-S-M (M stands for Ni or Fe) bond in Ir_1_/NFS, further confirming the charge transfer from Ir atoms to the substrate (Fig. 2c). Notably, SO_x_^2−^ species are observed in the S 2*p* spectra, indicating that the surface sulfur element is oxidized to some extent. The Ni^2+^ 2*p* and Fe^3+^ 2*p* XPS spectra of Ir_1_/NFS have no shift compared with that in NFS (Figs. 2d, e). Moreover, we further studied the charge transfer between other SACs and the sulfide substrate, including Ru_1_/NFS, Au_1_/NFS, and Pt_1_/NFS. It is observed that the valence states of Ru, Au, and Pt in Ru_1_/NFS, Au_1_/NFS, and Pt_1_/NFS are Ru^3+^ [463.3 eV (4*f*_7/2_)], Au^3+^ [83.9 eV (4*f*_7/2_)], and Pt^4+^ [72.8 eV (4 *f*_7/2_)] based on the XPS spectra (Supplementary Figs. 20–22), suggesting that the charge transfer also exist between various precious metal atoms and the NFS substrate.

### Electrochemical oxygen evolution performance

**Fig. 3:**
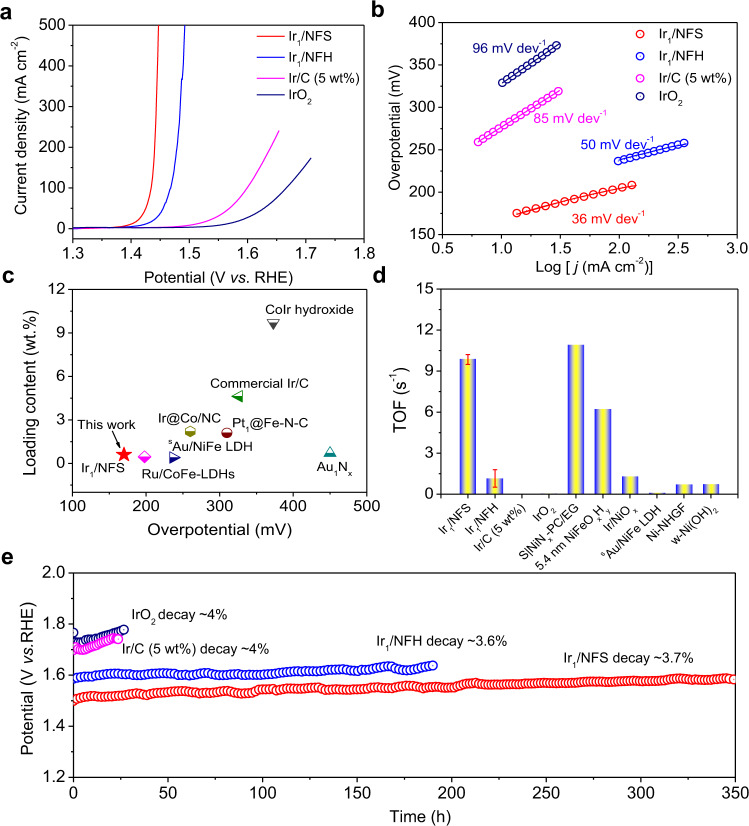
Electrochemical OER performance. **a** Polarization curves of Ir_1_/NFS, Ir_1_/NFH, Ir/C (5 wt%), and IrO_2_ in 1.0 M KOH. **b** Tafel plots derived from the polarization curves in (**a**). **c** The comparison of overpotentials and precious metal loading contents in reported catalysts at a current density of 10 mA cm^−2^. **d** The TOFs of state-of-the-art catalysts in alkaline solution (the error bars represent standard deviation values obtained from three independent measurements). **e** The stability tests of Ir_1_/NFS, Ir_1_/NFH, Ir/C (5 wt%), and IrO_2_ at 100 mA cm^−2^.

The electrocatalytic performances of the Ir_1_/NFS for OER were evaluated in 1.0 M KOH solution. All potentials were calibrated with respect to the reversible hydrogen electrode (RHE) (Supplementary Fig. 23). The commercial Ir/C (5 wt%) and IrO_2_ catalysts were also tested as a benchmark value for OER activity. Remarkably, the OER overpotential for Ir_1_/NFS is observed down to ~170 mV for reaching a current density of 10 mA cm^−2^, which is much lower than Ir_1_/NFH (~190 mV) and also the benchmark catalysts (Fig. 3a and Supplementary Table 1). Under more practical conditions, the OER overpotentials for Ir_1_/NFS are only 206 and 220 mV at current densities of 100 and 500 mA cm^−2^, respectively. In contrast, the Ir_1_/NFH demonstrates much higher overpotentials of 234 and 257 mV, respectively. We furthe investigated the substrate effect on the OER activity of Ir SAC by changing the ratio of Ni/Fe in the sulfide substrate (Supplementary Table 2). As shown in Supplementary Fig. 24, the Ir_1_/Ni_y_Fe_(6-y)_S_x_ (y = 0–6) electrodes have similar morphologies showing porous structures with interconnected wrinkles. The electrocatalytic activity of the Ni_y_Fe_(6-y)_S_x_ (y = 0–6) substrate shows obvious Ni/Fe ratio dependence in 1.0 M KOH solution, among which the Ni_3_Fe_3_S_x_ exhibits the superior performance (Supplementary Fig. 25). The Ir_1_/NFS catalysts also follow the similar activity trend when changing the Ni/Fe ratio of NFS substrate (Supplementary Figs. 26 and 27). Tafel plots provide further insights into the OER kinetics (Fig. 3b). The Ir_1_/NFS has a very small Tafel slope (33 mV dec^−1^), which is lower than that of Ir_1_/NFH (35 mV dec^−1^), Ir/C (87 mV dec^−1^), and IrO_2_ (95 mV dec^−1^). The low Tafel slope of Ir_1_/NFS suggests that the NFS substrate could significantly improve the electrochemical kinetics of Ir single-atoms.

In order to exclude the geometric effects on activity assessment, the OER activities of various catalysts were also tested on a flat Ni film (Ni/Au_disc_), which was electrodeposited on a gold disc electrode. Ir_1_/NFS requires an overpotential of 230 mV for reaching a current density of 10 mA cm^−2^ on Ni/Au_disc_ electrode, while Ir_1_/NFH (258 mV) need relatively high overpotential to deliver the same current density (Supplementary Fig. 28). Additionally, the specific activity of Ir_1_/NFS and Ir_1_/NFH were calculated based on the mass of Ir loadings on the Ni/Au_disc_ electrode. The calculated specific activity for Ir_1_/NFS is 20.3 A g_Ir_^−1^ at 1.45 V versus RHE, which is ~1.55 times higher than that of Ir_1_/NFH (13.1 A g_Ir_^−1^). The specific activity of Ir_1_/NFS also outperforms most of the well-developed OER catalysts in literatures (Fig. 3c and Supplementary Table 4). The specific activity and turnover frequency (TOF) offers fundamental insights in evaluating the intrinsic activities of electrocatalysts^7,27^. The TOF was calculated based on the current density at the overpotential of 300 mV. The TOF of Ir_1_/NFS reaches up to 9.85 s^−1^, which is an order of magnitude higher than that of Ir_1_/NFH (1.15 s^−1^) and previously reported values in literatures (Fig. 3d and Supplementary Table 5).

The electrochemical impedance spectroscopy (EIS) was employed to investigate the charge transfer kinetics on various catalysts at an overpotential of 250 mV on Ni/Au_disc_. Besides the similar resistance of the electrolyte and electrode, the charge transfer resistance (R_ct_) for Ir_1_/NFS (~26.5 Ω) is much lower than that for Ir_1_/NFH (~83.1 Ω) based on the fitted value according to the equivalent circuit (Supplementary Fig. 29 and Supplementary Table 6). Furthermore, the electrochemical double-layer capacitances (C_dl_) was calculated to evaluate the electrochemically effective surface areas (ECSA) utilized a cyclic voltammetry method^42,43^. It is noted that the C_dl_ of Ir_1_/NFS (3.7 mF cm^−2^) is ~2.0 times greater than that of the Ir_1_/NFH (1.3 mF cm^−2^), manifesting that Ir_1_/NFS possesses the most catalytic active sites for OER (Supplementary Fig. 30).

The stability of catalyst is a critical parameter of OER performance, especially for practical applications. The chronoamperometry method was utilized to estimate the stability of Ir_1_/NFS and other samples at an overpotential of 250 mV on Ni foam electrodes. Strikingly, the Ir_1_/NFS displays a relatively higher and more stable current density (~50 mA cm^−2^) than that of Ir_1_/NFH (~27 mA cm^−2^) (Supplementary Fig. 31). Moreover, we evaluated the long-term electrochemical stability by using a chronopotentiometric technique at a relatively high current density of 100 mA cm^−2^. Ir_1_/NFS demonstrates a superior stability performance, showing only ~3.7% of activity decay after 350 h, which is much better than Ir_1_/NFH (~3.6% decay after 198 h), Ir/C (5 wt%) (~4% decay after 25 h) and IrO_2_ (~4% decay after 25 h) (Fig. 3e). To further investigate the leaching effect of the catalyst after a long-term OER test (100 h at 100 mA cm^−2^), the amounts of dissolution elements from Ir_1_/NFS into the electrolyte was detected by ICP-MS measurement. As shown in Supplementary Fig. 32, the Ir content in electrolyte for Ir_1_/NFS electrode is only ~1 ppb, indicating that the Ir element is stable in the Ir_1_/NFS catalyst during OER test. On the other hand, the Ni, Fe, and S content in electrolyte for Ir_1_/NFS electrode is determined to be ~5 ppb, ~13 ppb, and ~98 ppb, respectively, implying that the substrate transformation corrosion take place during the OER test. To further demonstrate the overall electrochemical water splitting, a large-area Ir_1_/NFS electrode (20 cm × 30 cm) was prepared (Supplementary Fig. 33), which was used to build up a two-electrode electrolysis device for water electrolysis. NiFe-OH-PO_4_ was used as hydrogen evolution catalyst in this device which was synthesized according to the previous literature^44^. As shown in Supplementary Fig. 34, the water electrolysis process could be driven by a single-cell AAA battery with a nominal voltage of ~1.5 V (Supplementary Movie 1).

In addition, the morphology characterizations of the post-OER Ir_1_/NFS show that the initial nanosheet arrays with interconnected wrinkles are still maintained after testing for 10 h at an overpotential of 250 mV (Supplementary Fig. 35). The TEM images reveal that the post-OER Ir_1_/NFS nanosheets are composed of a fluffy ultrathin layered structure (Supplementary Fig. 36). The SAED patterns of the post-OER Ir_1_/NFS showed two diffraction rings, indicating an amorphous structure transformation after the OER test. The resulting EDX mapping images (Supplementary Fig. 37) show that Ni, Fe, Ir, S, and O are still homogeneously distributed over the entire post-OER Ir_1_/NFS sample. In addition, the HAADF-STEM images exhibit that the monoatomic Ir atoms (bright dots) uniform disperse on the surface of substrate and have no obvious aggregated clusters (Supplementary Figs. 38 and 39). Compared with the initial Ir_1_/NFS, the crystal structure post-OER Ir_1_/NFS shows a poor crystalline structure, but the main peaks of NFS can be detected as shown in Supplementary Fig. 40. In addition, as shown in the Supplementary Fig. 41, the Ir-S-M (M stands for Ni or Fe) bond is still maintained in the post-OER Ir_1_/NFS according to the S 2*p* spectrum. It is noted that the binding energy of Ir^3+^ 4*f*_7/2_ in post-OER Ir_1_/NFS has a negatively shift (~0.1 eV) compared with that in Ir_1_/NFS, indicating the coordination change occur during the OER test. The XPS spectra of S 2*p* in post-OER Ir_1_/NFS show that the peak strength ratio of S^2−^ and SO_x_^2−^ displays some predictable changes, implying that the surface sulfur element of NFS is oxidized to some extent^45–47^.

### Mechanistic understanding on substrate effect on Ir SAC

**Fig. 4:**
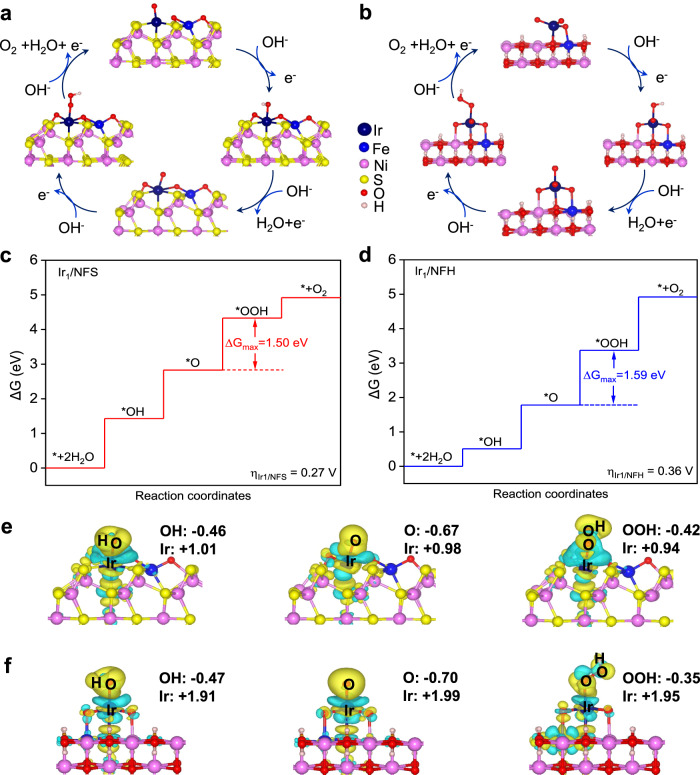
DFT theoretical models. **a**, **b** Proposed 4e^−^ mechanism of oxygen evolution reaction on Ir_1_/NFS and Ir_1_/NFH. **c**, **d** The free energy profiles of four-step elementary reaction on Ir_1_/NFS and Ir_1_/NFH. **e**, **f** Diagrams of charge transfer between oxygen-containing intermediates and Ir atom during the oxygen evolution reaction of (**e**) Ir_1_/NFS and (**f**) Ir_1_/NFH. Green area represents the loss of electrons, while yellow area represents the opposite.

To further study the different OER activities on Ir_1_/NFS and Ir_1_/NFH structure models, the spin-polarized density functional theory calculations were employed to simulate the OER process based on the 4 e^−^ mechanism proposed by Nørskov (Fig. 4a, b)^48,49^. The Gibbs free energy profiles were calculated to investigate the nature of the activity difference between Ir_1_/NFS and Ir_1_/NFH and the free energy change of each elementary step is listed in Supplementary Table 7. The rate-determining step towards OER for the loaded Ir atom is the conversion of *O to *OOH (Fig. 4c, d), consistent with the previous studies under alkaline conditions^50,51^. In comparison with the limiting potential of 1.59 V versus computed hydrogen electrode (CHE) for Ir_1_/NFH’s rate-determining step, the loaded Ir atom on Ir_1_/NFS possesses a better activity with the significantly lower limiting potential of 1.50 V versus CHE. This result implies that the replacement of the substrate from oxide to sulfide can efficiently reduce the limiting potential and greatly enhance OER activity. As shown in Supplementary Figs. 43 and 44, the additional theoretical analysis of the NFS without Ir SAC emphasizes that the Ir SAC is the main factor for the exceptional alkaline OER catalysis of the Ir_1_/NFS.

Then we calculated the electronic properties of Ir atoms on different substrates to get more deep insights into the effect of substrate on the OER catalytic performance. To get more deep insights into the effect of substrate on the OER catalytic performance, we calculated the electronic properties of Ir atoms on different substrates. The total density of states (TDOS) (Supplementary Fig. 45) shows that Ir_1_/NFS has more electronic states near the Fermi level compared to Ir_1_/NFH, resulting in higher electrical conductivity, which is consistent with the results of resistance test. As the active site for adsorbing different intermediates, the electronic properties of the 4*d* orbitals of the Ir atom are of concern to us. The projected density of states (PDOS) results (Supplementary Figs. 46 and 47) suggest that the electrons on Ir atoms in Ir_1_/NFS are closer to the Fermi level, and tend to be delocalized throughout the reaction compared with Ir_1_/NFH, which contributes to the enhanced electrochemical performance because of the elevated electron transfer.

Furthermore, the differential charge density of the surface of Ir_1_/NFS and Ir_1_/NFH was adopted to rationalize the OER activity difference observed in the experiments. Combined with Bader charge analysis^52,53^, we have thoroughly studied the charge transfer between Ir atoms and different intermediates (Fig. 4e, f, and Supplementary Table 8). Compared with oxide substrates, Ir atoms on sulfide substrates tend to lose fewer electrons and exhibit a lower valence, which leads to the relatively weaker coupling of Ir atoms and oxygen-containing intermediates (Supplementary Table 9). There is still a linear relationship between the adsorption Gibbs free energy of intermediates *O and *OOH on Ir_1_/NFS (2.90 eV) and Ir_1_/NFH (2.86 eV). Charge transfer from substrate to form *O/Ir_1_ from *OH/Ir_1_ and the back transfer from *O/Ir_1_ to form *OOH/Ir_1_ is closer to each on Ir_1_/NFS than Ir_1_/NFH, which indicates that the replacement of the substrates is just conducive to the equal distribution of the reaction Gibbs free energies of the transfer from *OH to *O and *O to *OOH and lowers the rate-limiting potential. Meanwhile, we considered the oxidation of the substrate near iridium atoms to simulate the influence of the oxidizing environment (Supplementary Figs. 48 and 49). According to our results, the theoretical overpotential increased to varying degrees according to the number of oxygen atoms replaced. Comparing these overpotentials, conclusion can be drawn that the excellent catalytic activity exhibited by Ir_1_/NFS is closely related to the loading of iridium atoms on sulfur atoms. The oxidation of the substrate may destroy the chemical environment around the iridium atoms, which on the other hand demonstrates the superiority of the sulfide substrate. Therefore, both theoretical and experimental results are in agreement that the OER performance could be increased by dispersing Ir on NFS support with strong synergetic coupling which significantly enhanced intrinsic electrocatalytic activity and stability.

## Discussion

In summary, the Ir_1_/NFS was synthesized via a general strategy to anchor a low mass loading but high distribution density Ir single-atom sites on the NFS support by a two-step electrochemical method. Compared with Ir_1_/NFH, Ir_1_/NFS reveals an excellent OER performance in alkaline electrolyte. Based on DFT calculations, the superior activity could be attributed to the Ir-S moiety in Ir_1_/NFS, manifested in favorable formation of the *OOH intermediate in the OER process. Meanwhile, the weak interaction between isolated Ir atoms and NFS is optimal to proceed OER compared with that Ir-O-M bond in Ir_1_/NFH, avoiding formation of the high oxidation state of Ir in Ir_1_/NFS. This work can help us understand the interaction of the sulfide substrate and precious metal single-atom catalysts for water oxidation, which may also inspire further work in catalyst design in the broad high concentration and stability of single atomic catalysis area.

## Methods

### Synthesis of Ir_1_/NFS

Ni foam (NF, thickness: 0.5 mm) was sonicated in 5.0 M HCl solution to remove surface oxide layer. Then, the Ni foam was washed with water and acetone, subsequently, dried at 40 °C under vacuum as electrode. Ni-modified gold electrode (Ni/Au_disc_) was prepared by electrodeposition on Au electrode (*ϕ* = 5 mm) in electrolyte containing 1.0 M NiSO_4_·6H_2_O and 2.0 M Orthoboric acid at 60 °C. The current density of the prepared Ni/Au_disc_ is 2.5 mA cm^−2^ for 1000 s under 600 rpm.

A standard three-electrode electrochemical cell was used as the electrodeposition device which containing Ni foam and Ni/Au_disc_ as the working electrodes, a carbon rod as the counter electrode and a Hg/HgO electrode (1.0 M KOH) as the reference electrode. Ir_1_/NFS electrode was prepared by a two-step electrodeposition method. The electrolyte was composed of 3 mM Ni(NO_3_)_2_·6H_2_O, 3 mM Fe(NO_3_)_3_·9H_2_O and 3 mM thiourea. The cyclic voltammetry (CV) was carried out in the potential range from 0.2 V to −1.2 V versus Hg/HgO at 5 mV s^−1^ for three cycles to get the NFS substrate. Then, 0.05 mM precursor (IrCl_3_·xH_2_O) was added to the electrolyte and the potential range was changed to 0.3 V to −0.3 V versus Hg/HgO at 50 mV·s^−1^ for 15 cycles to get the Ir_1_/NFS. Besides Ni foam electrode, Ir_1_/NFS catalyst was also electrodeposited on Ni/Au_disc_ following the same procedures.

To verify the role of sulfide substrate, the Ir_1_/Ni_y_Fe_(6-y)_S_x_ (y = 0–6) electrodes with a variable Ni/Fe ratio were prepared by adjusting the Ni and Fe ions concentration in the electrodeposited electrolytes. The electrodeposition was followed by the same procedures as the Ir_1_/NFS electrode fabrication.

### Synthesis of M_1_/NFS (M = Pt, Au, Ru)

For the synthesis of Pt_1_/NFS, Au_1_/NFS, and Ru_1_/NFS, the precious precursors (H_2_PtCl_6_·6H_2_O, HAuCl_4_·4H_2_O, and RuCl_3_·xH_2_O) replaced IrCl_3_·xH_2_O in the electrolyte, respectively. The electrodeposition procedures were the same as these for the synthesis of Ir_1_/NFS.

### Synthesis of Ir_1_/NFH

For the synthesis of Ir_1_/NFH, thiourea was removed from the electrolyte and the electrodeposition procedures were the same as these for the synthesis of Ir_1_/NFS.

### Prepared the Ir/C and IrO_2_ electrodes

To prepare the Ir/C ink, 11 mg of Ir/C (5 wt% of Ir, Premetek Co.) was dispersed in 1 ml of water and ethanol solution (1:4, v/v), followed by the addition of 45 μl of Nafion 117 solution (Sigma-Aldrich). The mixture was then magnetic stirred to form a homogenous ink. Meanwhile, the IrO_2_ ink was also prepared, namely 5.14 mg of IrO_2_ and 5 mg of Ketjen Black dispersed in 1 ml of water and ethanol solution (1:4, v/v), followed by the addition of 40 μl of Nafion 117 solution (Sigma-Aldrich). The mixture was then sonicated briefly to form a homogenous ink. The ink was drop-casted onto the Ni foam and Ni/Au_disc_ electrodes and left dried in air.

### Characterizations

The morphologies and structures of as-prepared samples were characterized by field-emission scanning electron microscopy (SEM, Hitachi, SU8010, 10 kV) and transmission electron microscopy (TEM, Hitachi, H7700, 100 kV). HAADF-STEM images, and energy-dispersive X-ray (EDX) mappings were obtained by using a JEOL, JEM − 2100, 200 kV. X-ray diffraction (XRD) patterns were measured by using a Bruker D8 Advance X-ray diffractometer equipped with Cu Kα radiation (λ = 1.5418 Å). X-ray photoelectron spectra (XPS) were performed by using a Thermo Scientific ESCALAB 250Xi with monochromatic Al Kα X-ray sources (1486.6 eV) at 2.0 kV and 20 mA. Inductively coupled plasma mass spectrometer (ICP-MS) was performed on iCAP 7400. Raman spectra was carried out on a Renishaw RM 3000 Micro-Raman system.

### Electrochemical characterization

All electrochemical measurements were carried out with a three-electrode on the CHI 760E electrochemical workstation. As-prepared Ir_1_/NFS and Ir_1_/NFH electrodeposited on Ni foam (1 cm × 1 cm) and Ni/Au_disc_ electrodes were used directly as the working electrode without any further treatments. A carbon rod was used as the counter electrode and a Hg/HgO electrode (1.0 M KOH) was used as the reference electrode in 1.0 M KOH solution. All potential values in electrochemical measurements were calibrated with respect to the reversible hydrogen electrode (RHE). The calibration was performed in the high purity hydrogen 1.0 M KOH electrolyte with a Pt wire as the working electrode and a carbon rod as the counter electrode. The measured potentials versus Hg/HgO were converted to the values with reference to a RHE using the following equation^54^,

1 $${E}_{RHE}={E}_{Hg/HgO}+0.197\,V$$

Catalytic activity was assessed by linear sweep voltammetry (LSV) with a sweep rate of 5 mV·s^−1^ with 95% *iR* compensation. Tafel slopes were derived from the OER polarization curves obtained at 5 mV·s^−1^. For *iR* compensation, an *iR* compensation level of 95% was applied by using the automated *iR*-correction function of the potentiostat^55^. Electrochemical impedance spectroscopy (EIS) curves were recorded under amplitude 5 mV, and the frequency ranged from 10 K Hz to 0.1 Hz at an overpotential of 250 mV. The Double-layer capacitance (C_dl_) was determined by measuring the capacitive current associated with double-layer at non-Faradaic potential range charging from the scan-rate dependence of CV^43^. C_dl_ was estimated by plotting the half of the difference of the anodic and cathodic current density against the scan rate. All chronopotentiometry and chronoamperometry measurements were conducted under the same experimental setup without *iR* compensation.

Overall water splitting test condition: a two-electrode electrolysis device was fabricated by using Ir_1_/NFS as the OER catalyst and NiFe-OH-PO_4_ as hydrogen evolution catalyst in 1.0 M KOH solution. The NiFe-OH-PO_4_ was electrodeposited onto the Ni foam by CV in electrolyte containing 3.0 mM NiCl_2_·6H_2_O, 3.0 mM FeCl_2_·4H_2_O, and 1.0 mM NaH_2_PO_2_. The CV curves were carried out in the potential range from 0.2 V to −1.2 V versus Hg/HgO at 5 mV s^−1^ for three cycles to get the NiFe-OH-PO_4_ catalyst. Subsequently, the Ir_1_/NFS and NiFe-OH-PO_4_ catalysts were assembled into a two-electrode system, and the electrodes area were 2 cm × 2 cm, respectively. The overall water splitting was driven by a single-cell AAA battery with a nominal voltage of ~1.5 V in 1.0 M KOH.

### Calculation of turnover frequency (TOF)

The TOF values of Ir_1_/NFS, and Ir_1_/NFH coated on Ni/Au_disc_ were calculated according to the equation^56^,

2 $$TOF=j\times \frac{A}{4\times F\times m}$$

where *j* is the current density obtained at overpotential of 300 mV in A cm^−2^, and the contribution of the Ni and Fe active sites to the current density is deducted. A is the surface area of the Ni/Au_disc_ electrode (0.19625 cm^2^), F is the Faraday efficiency (96,485 C mol^−1^) and m is the number of moles of the Ir deposited onto the Ni/Au_disc_ electrode which was calculated by ICP-MS measurement.

### Theoretical calculations

In this study, all the spin-polarized calculations were performed by using the Vienna Ab-initio Simulation Package (VASP) with the projector augmented wave method for the core region and a plane-wave kinetic energy cutoff of 400 eV^57–59^. The DFT + U calculations were performed in calculating the nickel oxide system, while the value of U was 4.3 for Fe and 3.8 for Ni according to the previous studies^60^. The generalized gradient approximation method with Perdew-Burke-Ernzerh of (PBE) functional for the exchange-correlation term was used^61^. The models we adopted for calculation were modified from the surface of Ni_3_S_2_ (003)^62^ and Ni(OH)_2_ (012), which are based on XRD and SADE results. After replacing the Ni atom with a Fe atom at the outermost layer, an Ir atom was loaded on S atoms or O atoms near it. In order to simulate the environment where oxygen is enriched on the electrode surface in actual electrocatalytic process, both Ir and Fe atoms have been highly coordinated by O atoms as shown in Fig. 4a and Supplementary Fig. 42. Ir_1_/NFS model used in our calculation consists of 12.21 Å × 12.21 Å × 25.18 Å and Ir_1_/NFH consists of 9.31 Å × 1.93 Å × 22.06 Å. The large vacuum region was set at least 15 Å in *z* direction for the isolation of surface to prevent the interaction between two periodic units. A 3 × 3 × 1 Monkhorst-Pack sampled k-point grid was used to sample the reciprocal space. For the structure optimization, the convergence of energy and forces on each atom were set to be less than 1 × 10^−5^ eV and 0.02 eV/Å. During our calculation, top two layers were fully relaxed and the other layers are fixed at the lattice positions.

According to the OER cycle proposed by Nørskov^49^, the OER reaction follows the four-electron mechanism, corresponding to the four primitive steps listed in Eqs. (3) to (6), which involve adsorbed OH, O, and OOH intermediates on the surface (*),

3 $$\ast +{{{{{{\rm{OH}}}}}}}^{-}\to \ast {{{{{\rm{OH}}}}}}+{{{{{{\rm{e}}}}}}}^{-}$$

4 $$\ast {{{{{\rm{OH}}}}}}+{{{{{{\rm{OH}}}}}}}^{-}\to {{{{{{\rm{H}}}}}}}_{2}{{{{{\rm{O}}}}}}+\ast {{{{{\rm{O}}}}}}+{{{{{{\rm{e}}}}}}}^{-}$$

5 $$\ast {{{{{\rm{O}}}}}}+{{{{{{\rm{OH}}}}}}}^{-}\to \ast {{{{{\rm{OOH}}}}}}+{{{{{{\rm{e}}}}}}}^{-}$$

6 $$\ast {{{{{\rm{OOH}}}}}}+{{{{{{\rm{OH}}}}}}}^{-}\to {{{{{{\rm{O}}}}}}}_{2}+{{{{{{\rm{H}}}}}}}_{2}{{{{{\rm{O}}}}}}+{{{{{{\rm{e}}}}}}}^{-}$$

The Gibbs free energy changes for these four elemental steps can be expressed respectively as^48^,

7 $$\varDelta {{{{{{\rm{G}}}}}}}_{1}={\mu }_{\ast {{{{{\rm{OH}}}}}}}-{\mu }_{\ast }-({\mu }_{O{H}^{-}}-{\mu }_{{e}^{-}})$$

8 $$\varDelta {{{{{{\rm{G}}}}}}}_{2}={\mu }_{\ast {{{{{\rm{O}}}}}}}-{\mu }_{\ast {{{{{\rm{OH}}}}}}}+{\mu }_{{H}_{2}O(l)}-({\mu }_{O{H}^{-}}-{\mu }_{{e}^{-}})$$

9 $$\varDelta {{{{{{\rm{G}}}}}}}_{3}={\mu }_{\ast {{{{{\rm{OOH}}}}}}}-{\mu }_{\ast {{{{{\rm{O}}}}}}}-({\mu }_{O{H}^{-}}-{\mu }_{{e}^{-}})$$

10 $$\varDelta {{{{{{\rm{G}}}}}}}_{4}={\mu }_{\ast }+{\mu }_{{H}_{2}O(l)}+{\mu }_{{O}_{2}(g)}-{\mu }_{\ast {{{{{\rm{OOH}}}}}}}-({\mu }_{O{H}^{-}}-{\mu }_{{e}^{-}})$$

where $\mu$ represents the chemical potentials of the indicated species.

Here, the difference of the chemical potentials of the ${{OH}}^{-}$ species and the electron can be described as,

11 $${\mu }_{O{H}^{-}}-{\mu }_{{e}^{-}}={\mu }_{{H}_{2}O(l)}-({\mu }_{{H}^{+}}+{\mu }_{{e}^{-}}\!)$$

Meanwhile, the chemical potential of the proton and electron is related to that of H_2_ according to the computational hydrogen electrode (CHE) approach.

12 $${\mu }_{{H}^{+}}+{\mu }_{{e}^{-}}=\frac{1}{2}{\mu }_{{H}_{2}(g)}-e{U}_{RHE}$$

where ${U}_{{RHE}}$ was the potential of the electrode relative to the RHE. Thus, Eqs. (13)–(16) can be rewritten as,

13 $$\varDelta {{{{{{\rm{G}}}}}}}_{1}={\mu }_{\ast {{{{{\rm{OH}}}}}}}-{\mu }_{\ast }-{\mu }_{{H}_{2}O(l)}+\frac{1}{2}{\mu }_{{H}_{2}(g)}-e{U}_{RHE}$$

14 $$\varDelta {{{{{{\rm{G}}}}}}}_{2}={\mu }_{\ast {{{{{\rm{O}}}}}}}-{\mu }_{\ast {{{{{\rm{OH}}}}}}}-{\mu }_{{H}_{2}O(l)}+\frac{1}{2}{\mu }_{{H}_{2}(g)}-e{U}_{RHE}$$

15 $$\varDelta {{{{{{\rm{G}}}}}}}_{3}={\mu }_{\ast {{{{{\rm{OOH}}}}}}}-{\mu }_{\ast {{{{{\rm{O}}}}}}}-{\mu }_{{H}_{2}O(l)}+\frac{1}{2}{\mu }_{{H}_{2}(g)}-e{U}_{RHE}$$

16 $$\varDelta {{{{{{\rm{G}}}}}}}_{4}={\mu }_{\ast }-{\mu }_{\ast {{{{{\rm{OOH}}}}}}}+{\mu }_{{O}_{2}(g)}+\frac{1}{2}{\mu }_{{H}_{2}(g)}-e{U}_{RHE}\,$$

The chemical potential $\mu$ was defined as^63^ $\mu =E+{ZPE}-T\times S$, where *E*, *ZPE*, *T*, and *S* represented the total energy obtained from DFT calculations, the zero-point energy (*ZPE*), the temperature (298.15 K), the entropy obtained from vibrational frequency calculations, respectively.

The entropies of gas-phase H_2_, and H_2_O were obtained from the NIST database (Computational Chemistry Comparison and Benchmark Database. http://cccbdb.nist.gov/) with standard condition. Due to the non-negligible error in the first-principles calculation, the chemical potential of oxygen molecule is calculated as ${\mu }_{{O}_{2}}=2{G}_{{H}_{2}O(l)}-2{G}_{{H}_{2}}+4.92{eV}$. Then entropies of adsorbed species are considered according to vibrational entropy (${S}_{v}$) formula,

17 $${S}_{v}=\mathop{\sum}\limits_{i}R\left\{\frac{h{v}_{i}}{{k}_{B}T}{\left[{\exp }\left(\frac{h{v}_{i}}{{k}_{B}T}\right)-1\right]}^{-1}-{ln}\left[1-{\exp }\left(-\frac{h{v}_{i}}{{k}_{B}T}\right)\right]\right\}$$

All the real vibrational frequencies of the surface-adsorbing species were included to calculate the correction of the free energy.

Under ideal conditions, the OER reaction with a total energy change of 4.92 eV can be driven at 1.23 V, while the $\triangle$*G* of each elementary reaction would be equally divided into 1.23 eV. Therefore, the overpotential *η* is introduced to represent additional required potential and measure the catalytic activity of materials, which is defined in theoretical calculations as,

18 $${\eta} =\frac{{\max}[\varDelta {{{{{\rm{G}}}}}}_{1},{\varDelta} {{{{{\rm{G}}}}}}_{2},{\varDelta} {{{{{\rm{G}}}}}}_{3},{\varDelta} {{{{{\rm{G}}}}}}_{4}]}{e}-1{{{{{\rm{.23V}}}}}}$$

## Supplementary information

Supplementary Information

Description of Additional Supplementary Files

Supplementary Movie 1

## Data Availability

All the data supporting this study in the paper and Supplementary Information can be accessed using the link 10.6084/m9.figshare.17073017.v1. Source data are provided with this paper.

## Source data

Source Data
