# Supplementary material for: Coordination modulation of iridium single-atom catalyst maximizing water oxidation activity
Source: Nat Commun. 2022 Jan 10;13:24. doi: 10.1038/s41467-021-27664-z (PMC8748886; doi:10.1038/s41467-021-27664-z)
Supplement: Supplementary file 1 — Supplementary Information [file 41467_2021_27664_MOESM1_ESM.pdf]

Support information

Coordination Modulation of Iridium Single-Atom Catalyst Maximizing Water Oxidation Activity

Zhanwu Lei[1,5], Wenbin Cai[1,5], Yifei Rao[1,5], Kuan Wang[2], Yuyuan Jiang[2], Yang Liu[1], Xu Jin[3], Jianming Li[3], Zhengxing Lv[4], Shuhong Jiao[1,*], Wenhua Zhang[1,*], Pengfei Yan[2], Shuo Zhang[4], Ruiguo Cao[1,*]

1. Key Laboratory of Materials for Energy Conversion, Chinese Academy of Sciences (CAS), Department of Materials Science and Engineering, University of Science and Technology of China, Hefei, Anhui, 230026, China

2. Beijing Key Laboratory of Microstructure and Properties of Solids, Beijing University of Technology, Beijing, 100124, China

3. Research Center of New Energy, Research Institute of Petroleum Exploration and Development (RIPED), PetroChina, Beijing 100083, China

4. Shanghai Synchrotron Radiation Facility, Shanghai Institute of Applied Physics, Chinese Academy of Sciences, Shanghai 201204, China

5. These authors contributed equally.

Corresponding authors:

Shuhong Jiao: jiaosh@ustc.edu.cn

Wenhua Zhang: whhzhang@ustc.edu.cn

Ruiguo Cao: rgcao@ustc.edu.cn

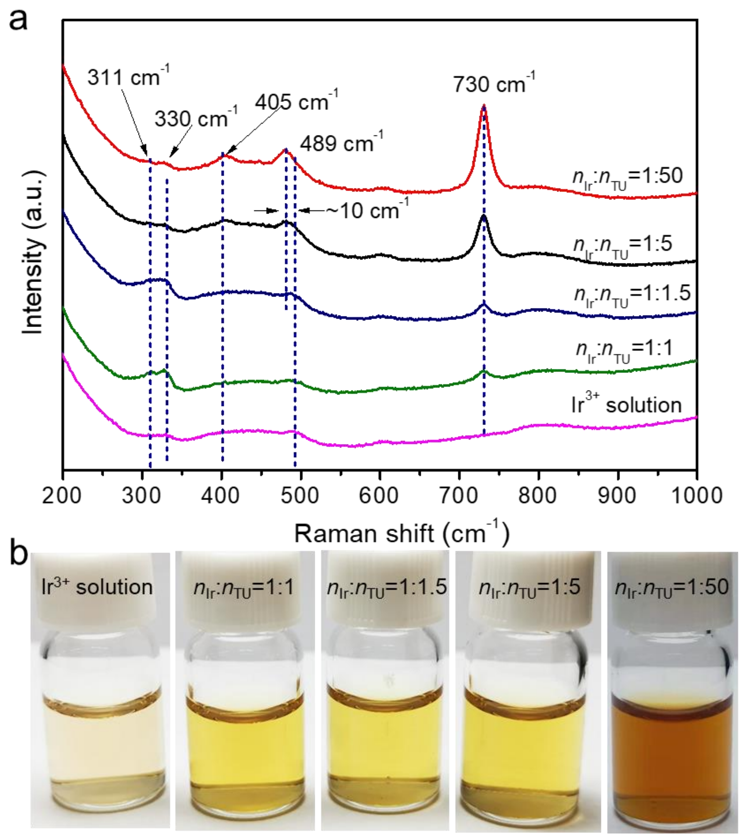

Supplementary Fig. 1 Raman spectra and the digital photographs of the solution with different molar ratios of n_{Ir} and TU. a. Raman spectra with different molar ratios of Ir^{3+} (n_{Ir}) and thiourea (TU) in solution. **b.** The digital photographs of the solution with different molar ratios of n_{Ir} and TU.

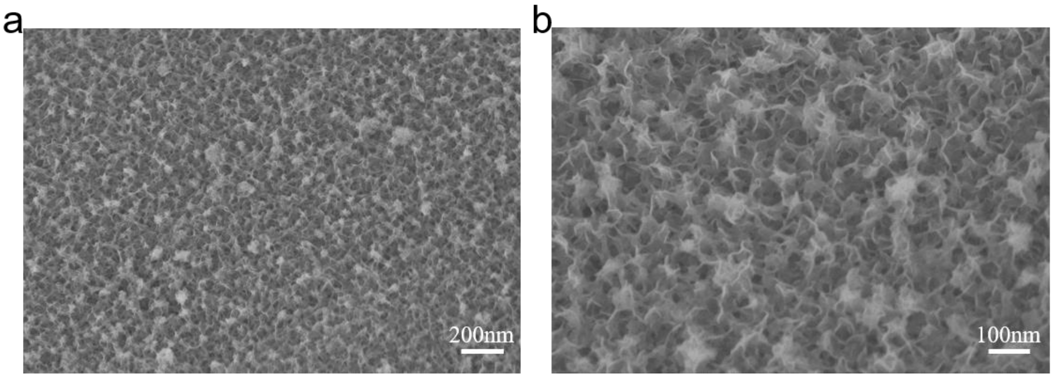

Supplementary Fig. 2 The morphology of Ir_1/NFS. SEM images of the as-prepared Ir_1/NFS. Scale bar, **a** 200 nm and **b** 100 nm.

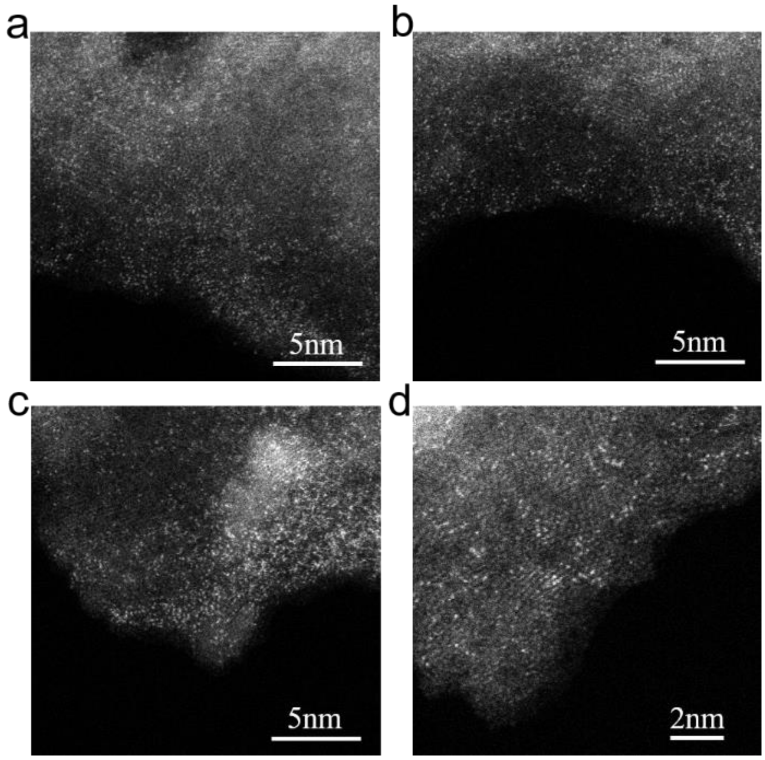

Supplementary Fig. 3 HAADF-STEM images of the as-prepared Ir_1/NFS. a - d High density bright dots corresponding to Ir single atoms are observed. Scale bar, **a** - **c** 5 nm and **d** 2 nm.

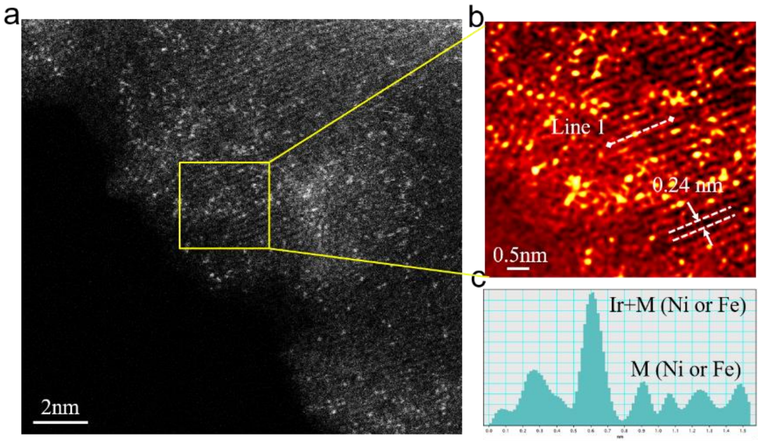

Supplementary Fig. 4 HAADF-STEM images of the as-prepared Ir_1/NFS. a and **b** The HAADF-STEM images of the Ir_1/NFS. **c** Intensity line profiles taken along the corresponding white line 1 in **b**. Scale bar, **a** 2 nm and **b** 0.5 nm.

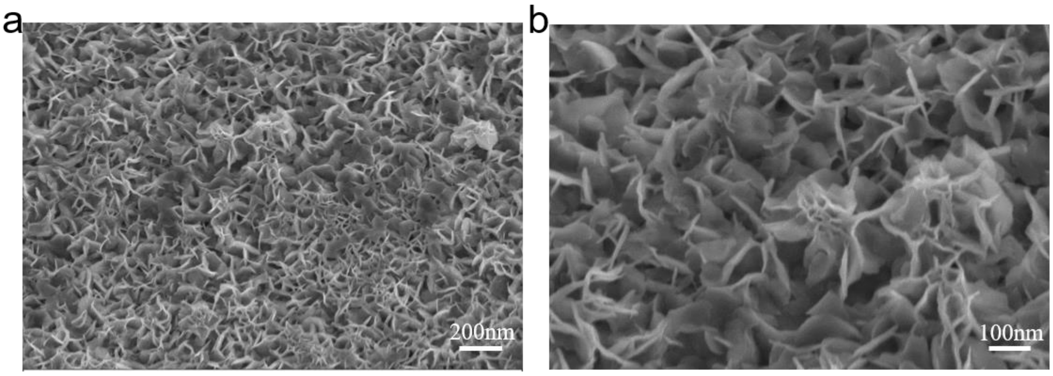

Supplementary Fig. 5 The morphology of Ir_1/NFH. SEM images of the as-prepared Ir_1/NFH. Scale bar, in **a** 200 nm and in **b** 100 nm.

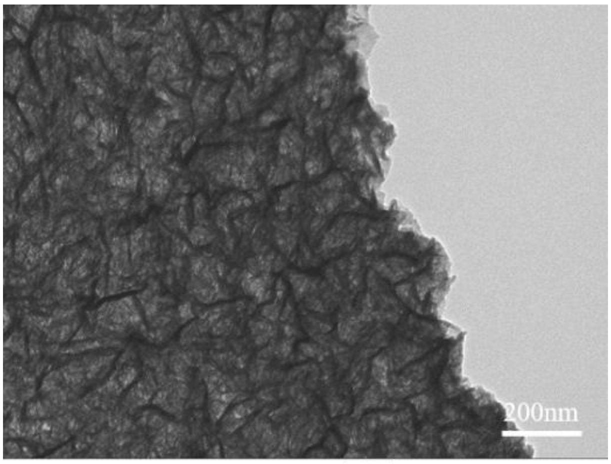

Supplementary Fig. 6 The morphology of Ir_1/NFH. TEM image of the as-prepared Ir_1/NFH. Scale bar, 200 nm.

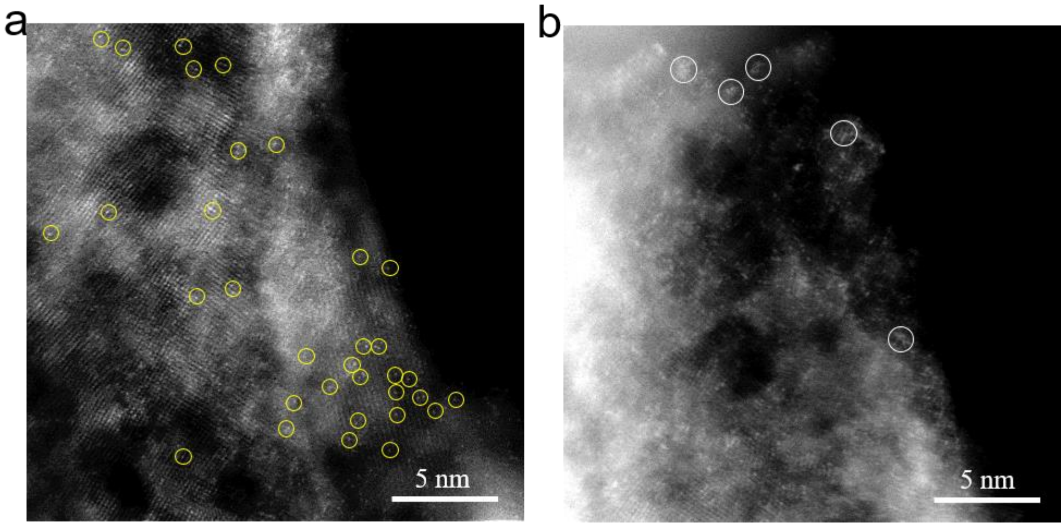

Supplementary Fig. 7 HAADF-STEM images of the as-prepared Ir_1/NFH. a High density bright dots (some of the isolated Ir atoms are marked by yellow circles) corresponding to Ir single atoms are observed. **b** A fewer Ir clusters are observed (some of the Ir clusters are marked by white circles). Scale bar, in **a** and **b** 5 nm.

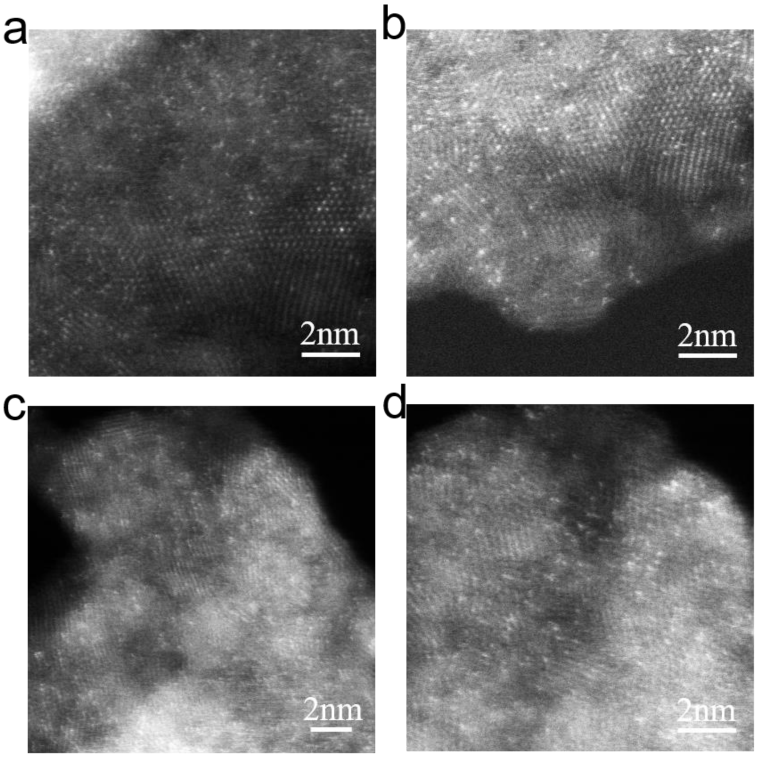

Supplementary Fig. 8 HAADF-STEM images of the as-prepared Ir_1/NFH. a - d High density bright dots corresponding to Ir single atoms are observed. Scale bar, **a - d** 2 nm.

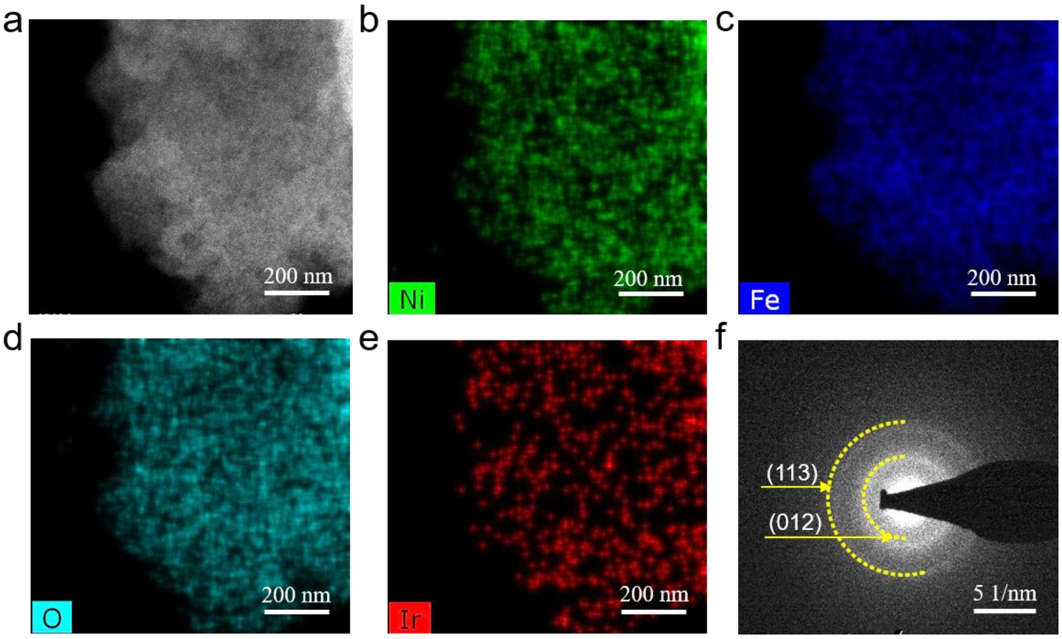

Supplementary Fig. 9 HAADF image and EDX elemental mappings of the as-prepared Ir_1/NFH. a HAADF image of the as-prepared Ir_1/NFH. **b-e** EDX elemental mappings of the as-prepared Ir_1/NFH. **f** The corresponding SADE of the as-prepared Ir_1/NFH. Scale bar, in **a-e** 200 nm and in **f** 5 1/nm.

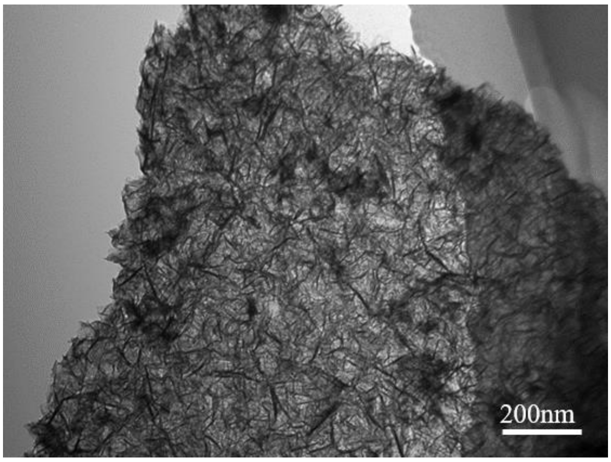

Supplementary Fig. 10 The morphology of Ru_1/NFS. TEM image of Ru_1/NFS. Scale bar, 200 nm.

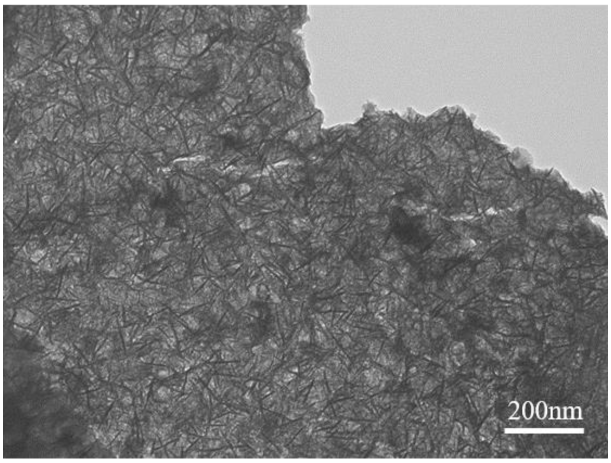

Supplementary Fig. 11 The morphology of Au_1/NFS. TEM image of Au_1/NFS. Scale bar, 200 nm.

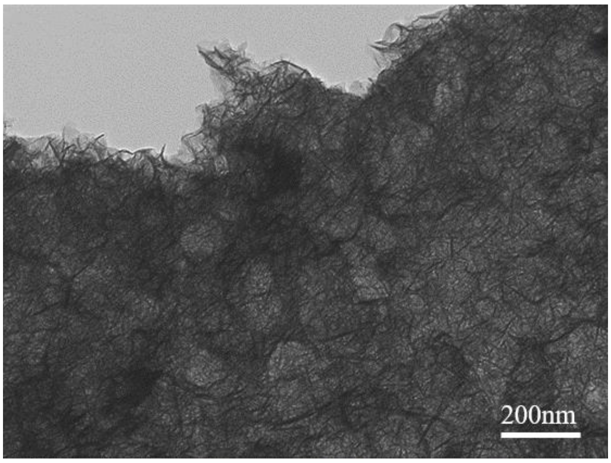

Supplementary Fig. 12 The morphology of Pt_1/NFS. TEM image of Pt_1/NFS. Scale bar, 200 nm.

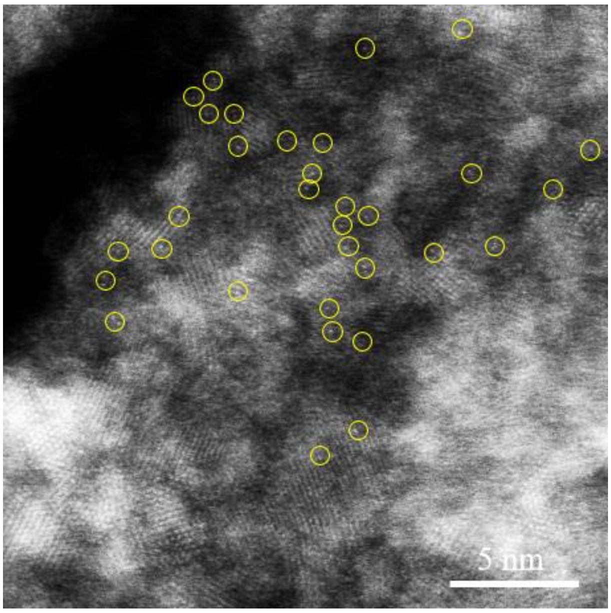

Supplementary Fig. 13 HAADF-STEM image of the as-prepared Ru_1/NFS. The bright dots (some of the isolated Ir atoms are marked by yellow circles) corresponding to Ir single atoms are observed. Scale bar, 5 nm.

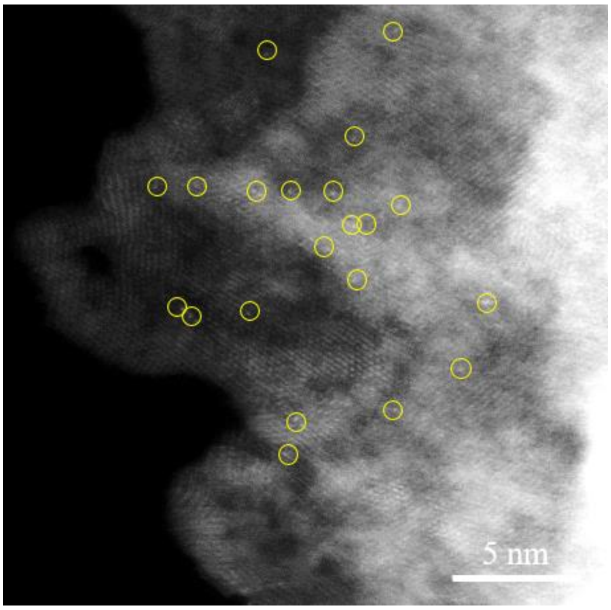

Supplementary Fig. 14 HAADF-STEM image of the as-prepared Au_1/NFS. The bright dots (some of the isolated Ir atoms are marked by yellow circles) corresponding to Ir single atoms are observed. Scale bar, 5 nm.

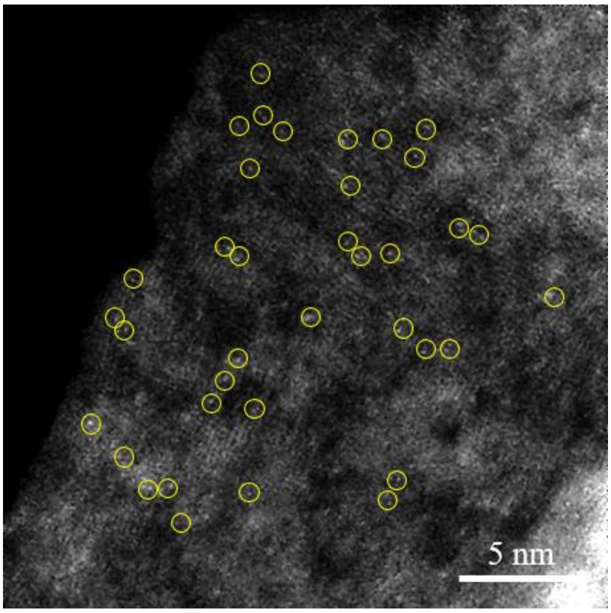

Supplementary Fig. 15 HAADF-STEM image of the as-prepared Pt_1/NFS. The bright dots (some of the isolated Ir atoms are marked by yellow circles) corresponding to Ir single atoms are observed. Scale bar, 5nm.

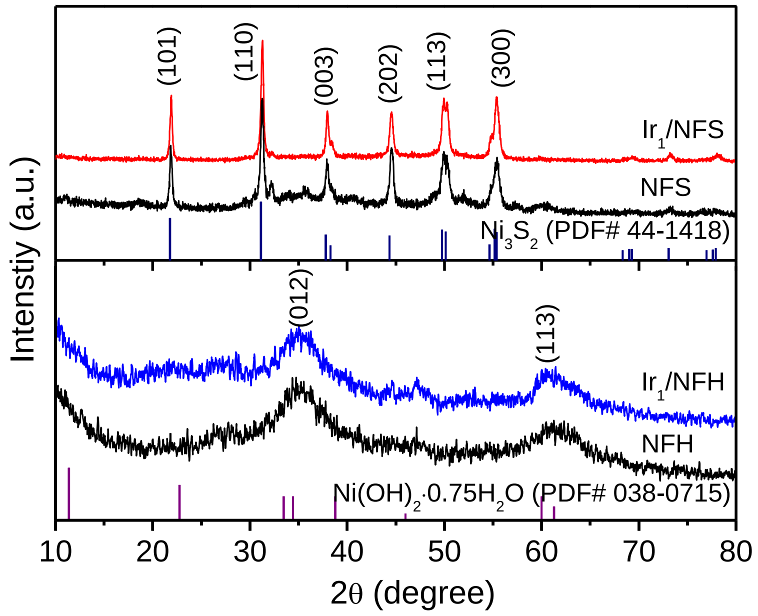

Supplementary Fig. 16 XRD characterizations. XRD patterns of the as-prepared Ir_1/NFS, NFS, Ir_1/NFH, and NFH, respectively.

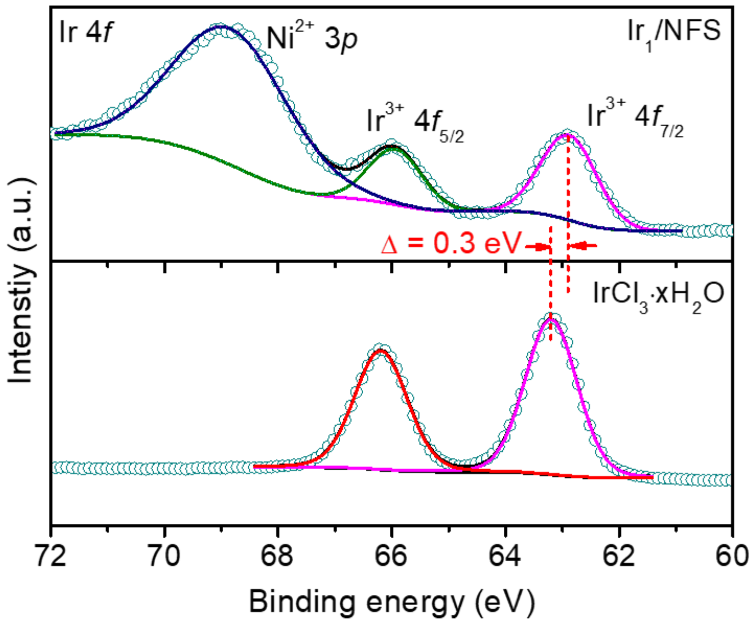

Supplementary Fig. 17 XPS characterizations. XPS spectra of Ir_1/NFS and $IrCl_3 \cdot xH_2O$ in the Ir 4*f* region.

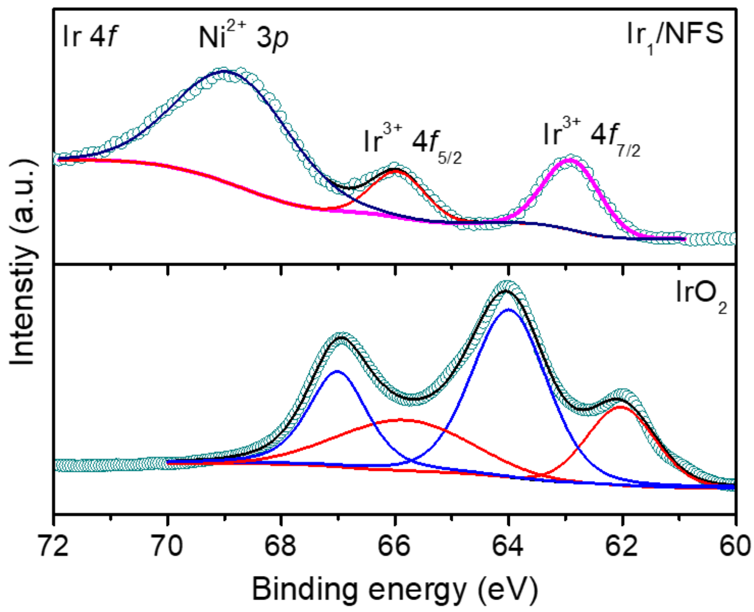

Supplementary Fig. 18 XPS characterizations. XPS spectra of Ir_1/NFS and IrO_2 in the Ir 4*f* region.

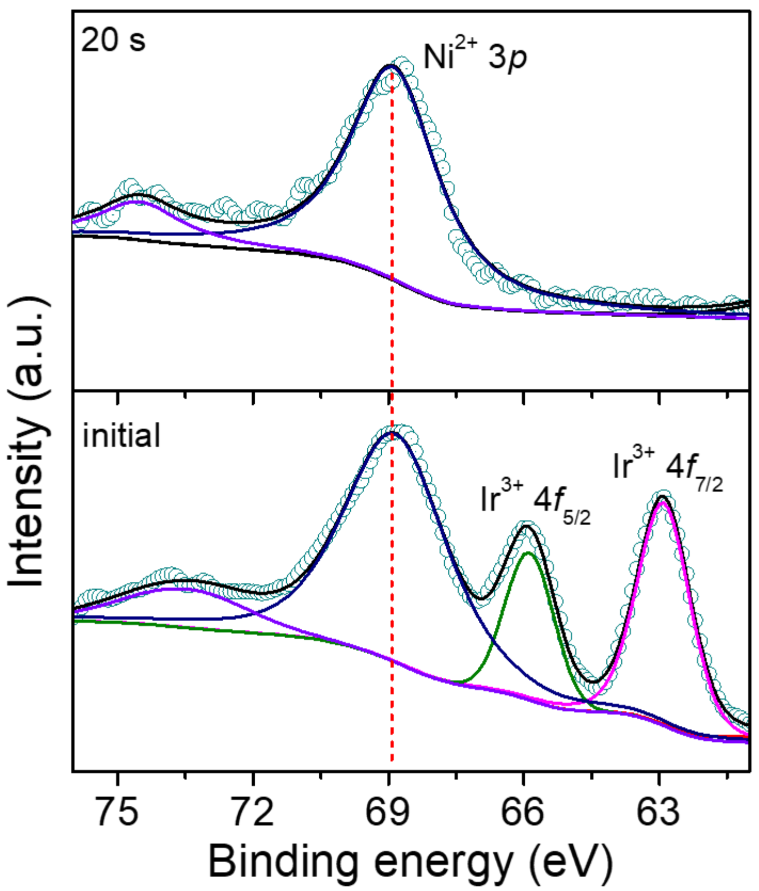

Supplementary Fig. 19 XPS characterizations. XPS depth profiling spectra of the Ir^{3+} 4*f* and Ni^{2+} 3*p* in Ir_1/NFS. XPS spectra were collected after etching Ar^+ for 0, and 20 s.

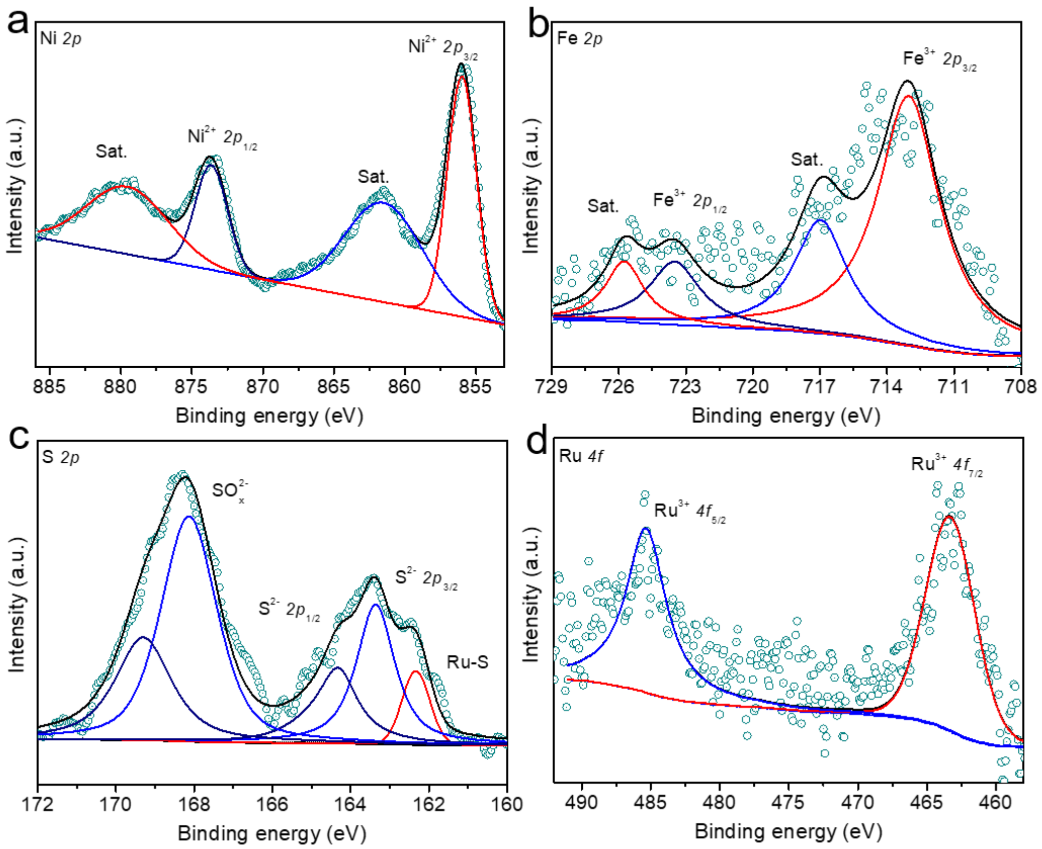

Supplementary Fig. 20 XPS characterizations of Ru_1/NFS. a – d XPS spectra of **a** Ni 2*p*, **b** Fe 2*p*, **c** S 2*p*, and **d** Ru 4*f* regions with fitting curves for Ru_1/NFS.

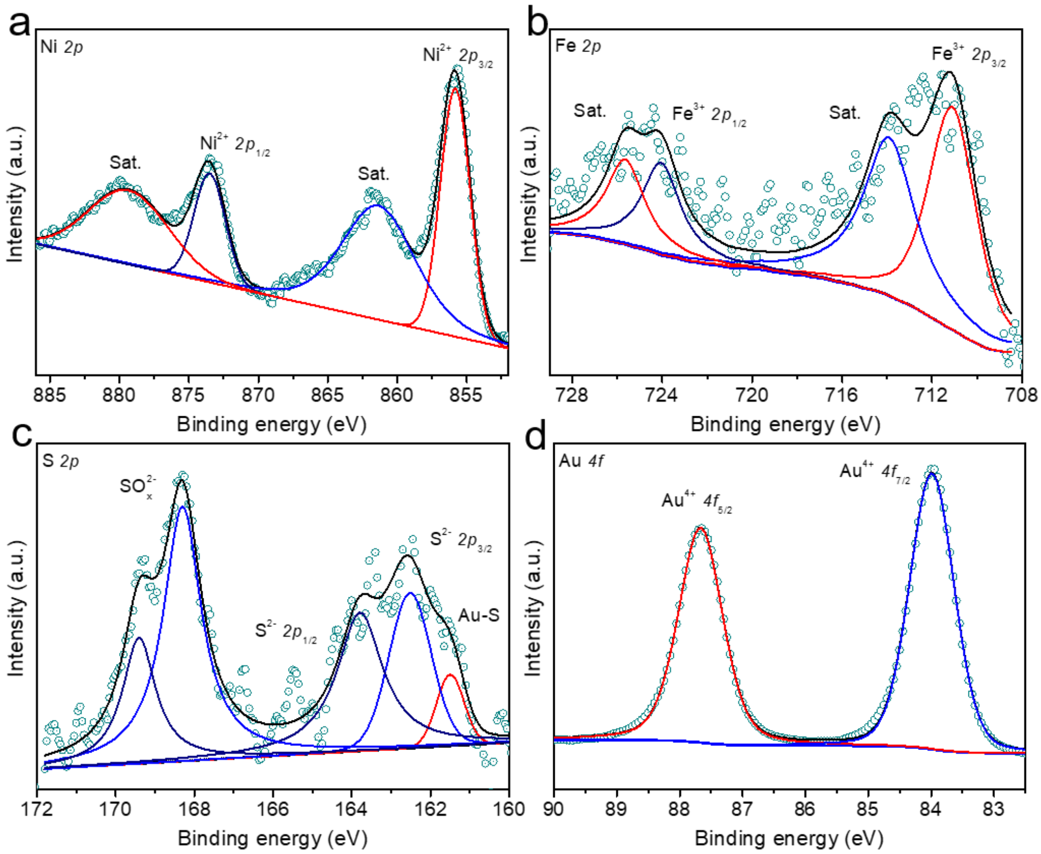

Supplementary Fig. 21 XPS characterizations of Au_1/NFS. a – d XPS spectra of **a** Ni 2*p*, **b** Fe 2*p*, **c** S 2*p*, and **d** Au 4*f* regions with fitting curves for Au_1/NFS.

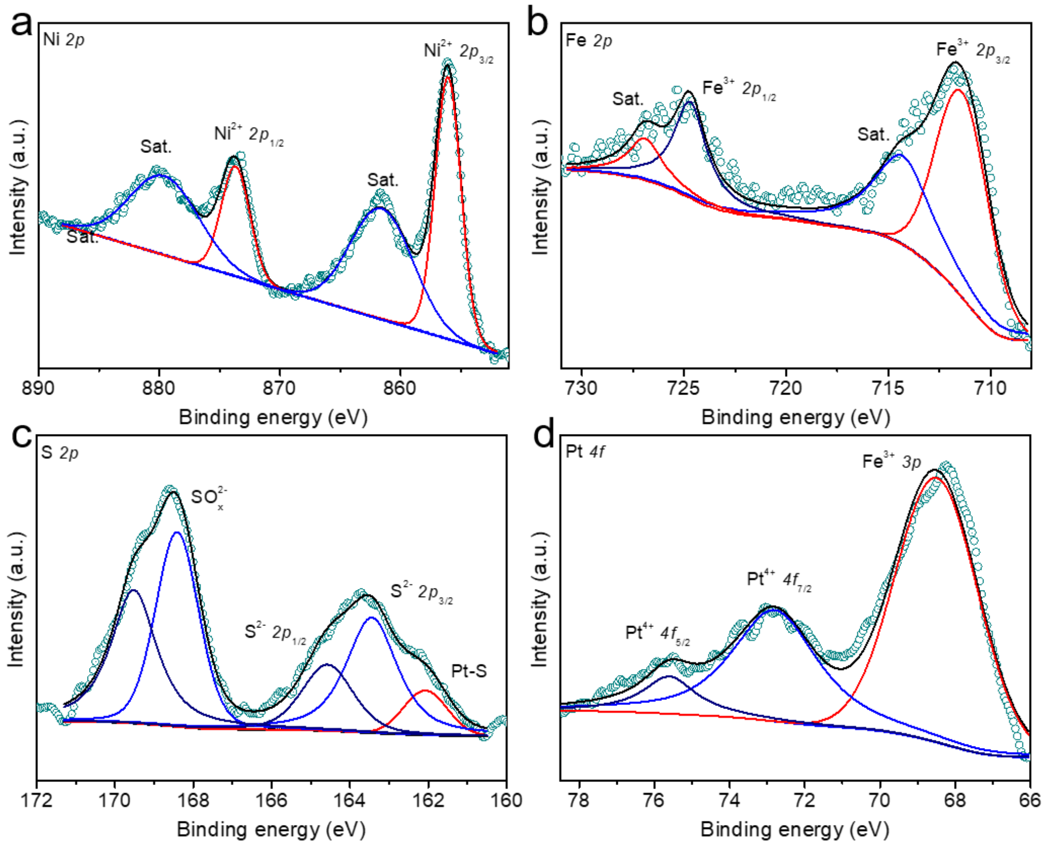

Supplementary Fig. 22 XPS characterizations of Pt$_1$/NFS. a – d XPS spectra of **a** Ni 2*p*, **b** Fe 2*p*, **c** S 2*p*, and **d** Pt 4*f* regions with fitting curves for Pt$_1$/NFS.

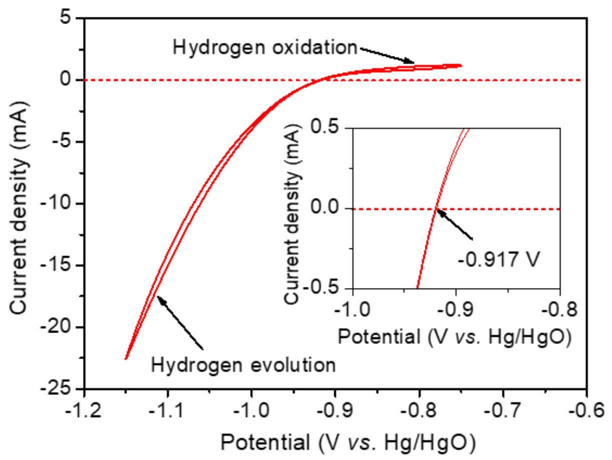

Supplementary Fig. 23 Calibration of reference electrodes. The Hg/HgO (1.0 M KOH) reference electrode was calibrated with respect to RHE in 1.0 M KOH.

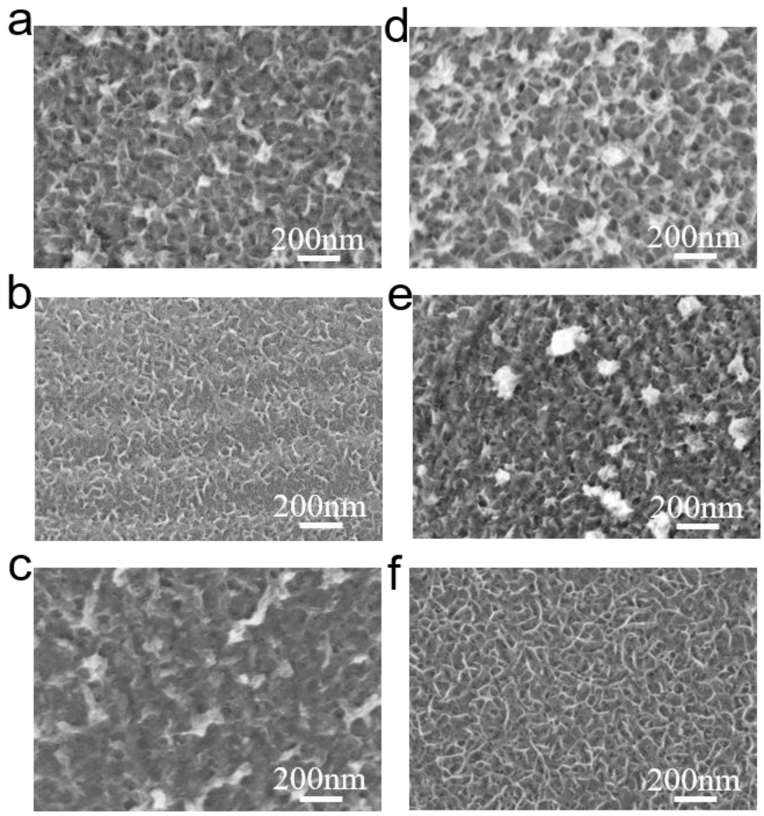

Supplementary Fig. 24 SEM images of $Ir_1/Ni_yFe_{(6-y)}S_x$ (y = 0 ~ 6) with the different relative ratio of Ni/Fe. **a** $Ir_1/Ni_0Fe_6S_x$, **b** $Ir_1/Ni_1Fe_5S_x$, **c** $Ir_1/Ni_2Fe_4S_x$, **d** $Ir_1/Ni_4Fe_2S_x$, **e** $Ir_1/Ni_5Fe_1S_x$, and **f** $Ir_1/Ni_6Fe_0S_x$. Scale bar, **a** - **f** 200 nm.

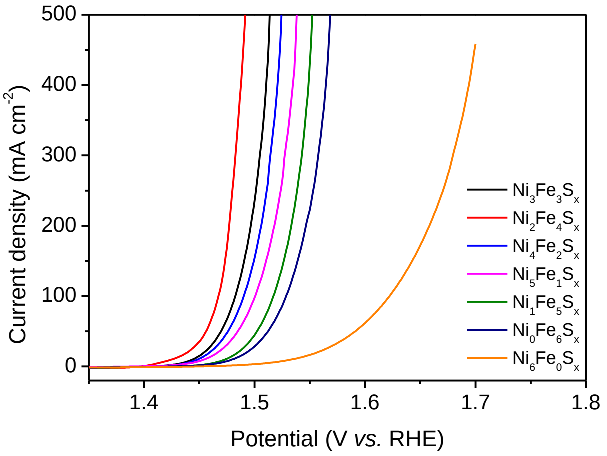

Supplementary Fig. 25 Electrochemical OER performance of $Ni_yFe_{(6-y)}S_x$ (y = 0 ~ 6). Polarization curves of $Ni_yFe_{(6-y)}S_x$ (y = 0 ~ 6) with the different relative ratio of Ni/Fe.

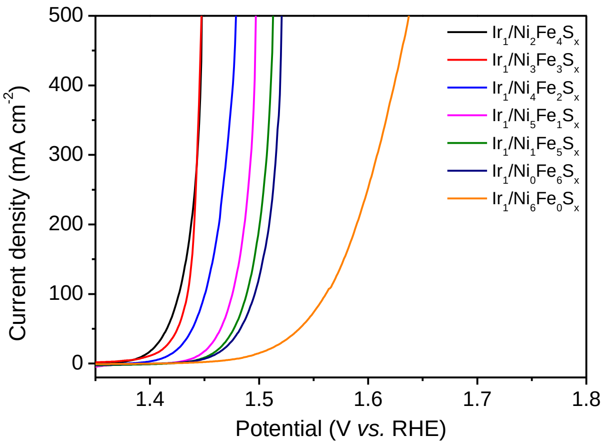

Supplementary Fig. 26 Electrochemical OER performance of $Ir_1/Ni_yFe_{(6-y)}S_x$ (y = 0 ~ 6). Polarization curves of $Ir_1/Ni_yFe_{(6-y)}S_x$ (y = 0 ~ 6) with the different relative ratio of Ni/Fe.

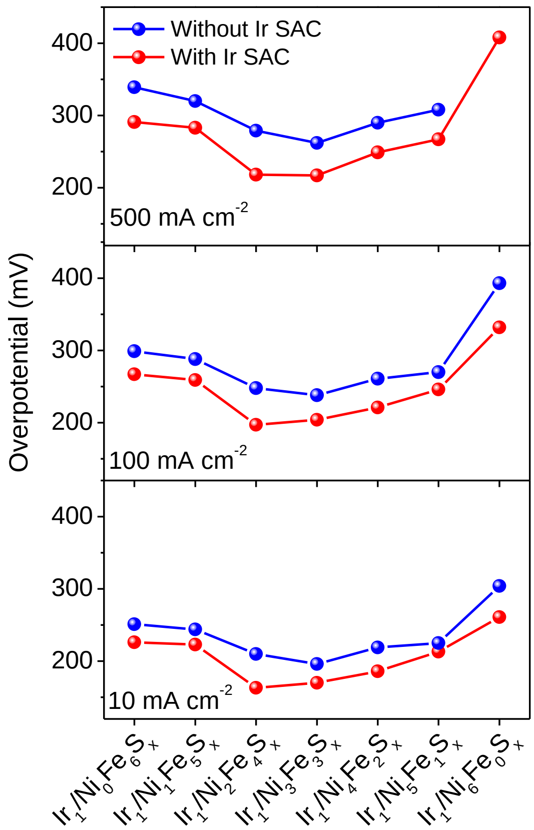

Supplementary Fig. 27 The overpotential of $Ir_1/Ni_yFe_{(6-y)}S_x$ (y = 0 ~ 6). The overpotential of $Ir_1/Ni_yFe_{(6-y)}S_x$ (y = 0 ~ 6) with/without Ir SAC at 10, 100, and 500 mA cm^{-2}.

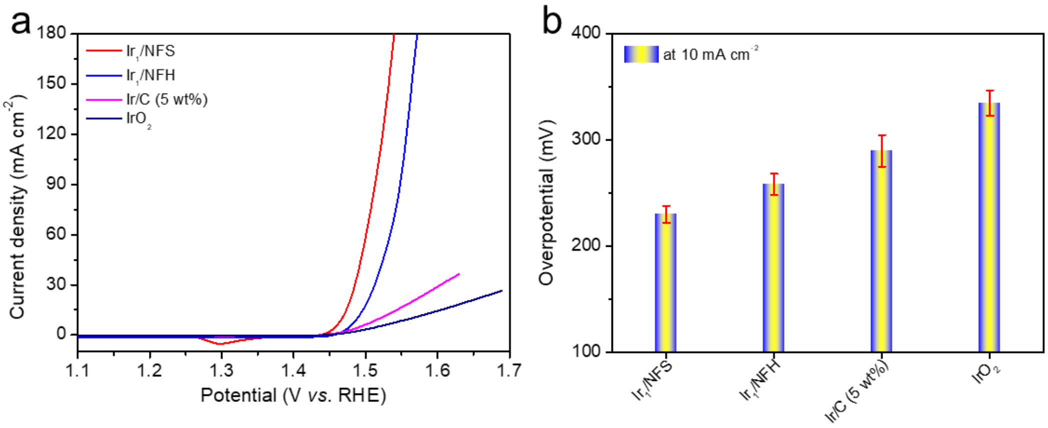

Supplementary Fig. 28 Electrochemical OER performance on Ni/Au$_{disc}$ electrode. **a** Polarization curves of Ir_1/NFS and various samples on Ni/Au$_{disc}$ measured in 1.0 M KOH solution. **b** The overpotentials of Ir_1/NFS and various samples on Ni/Au$_{disc}$ measured in 1.0 M KOH solution at 10 mA cm^{-2} (The error bars represent standard deviation values obtained from three independent measurements).

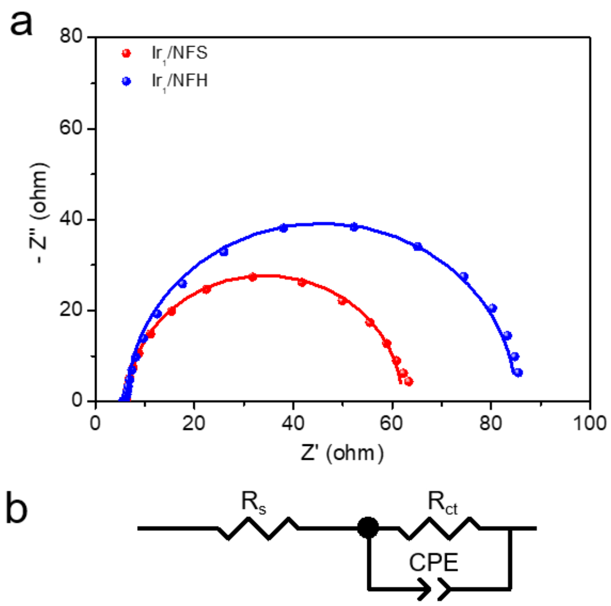

Supplementary Fig. 29 EIS of Ir_1/NFS, and Ir_1/NFH on Ni/Au_{disc} electrode. a EIS of Ir_1/NFS, and Ir_1/NFH at an overpotential of 250 mV on Ni/Au_{disc} electrodes. **b** The circuit diagram corresponding to EIS.

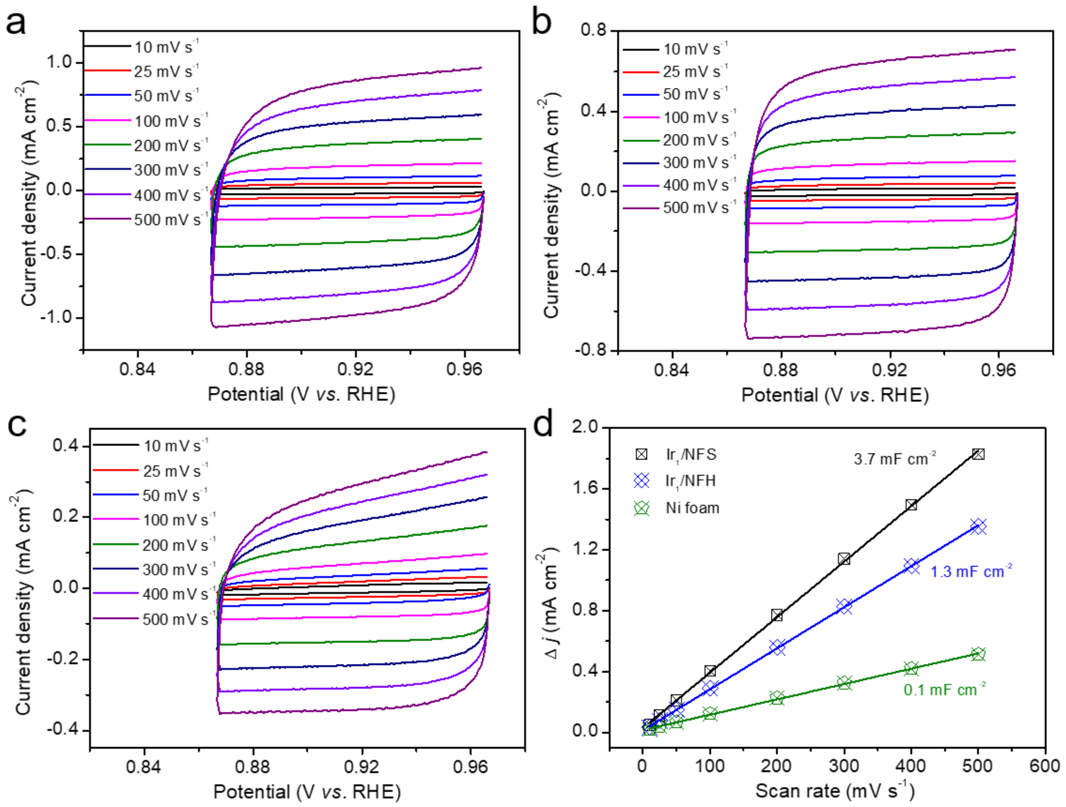

Supplementary Fig. 30 Double-layer capacitance measurements. a - c CVs at different scan rates of in a potential window where no Faradaic processes occur for **a** Ir_1/NFS, **b** Ir_1/NFH, and **c** Ni foam, respectively. **d** Charging current density differences at 0.91 V versus RHE plotted against scan rates. The linear slope is equivalent to the double-layer capacitance (C_{dl}).

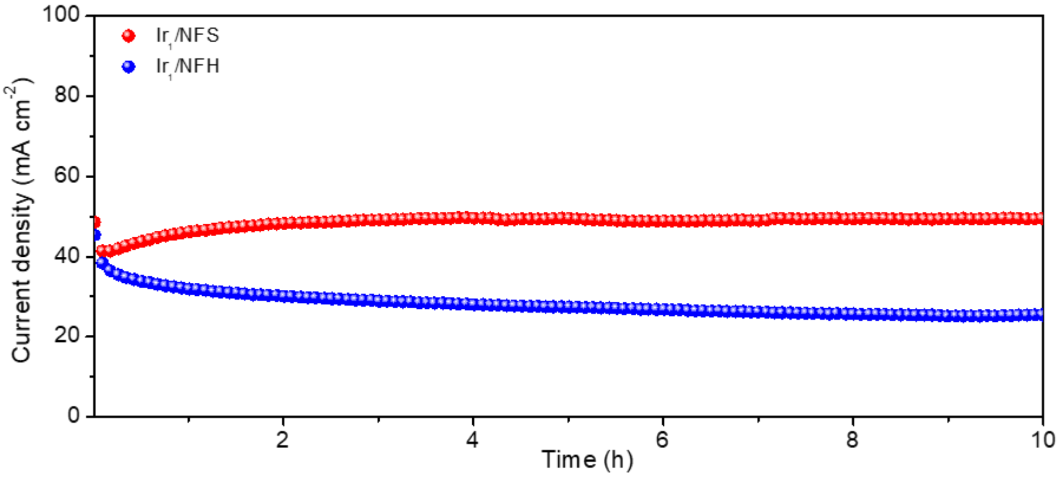

Supplementary Fig. 31 The stability tests of Ir1/NFS, and Ir1/NFH. Chronoamperometry measurements of Ir1/NFS, and Ir1/NFH at an overpotential of 250 mV on Ni foam electrodes.

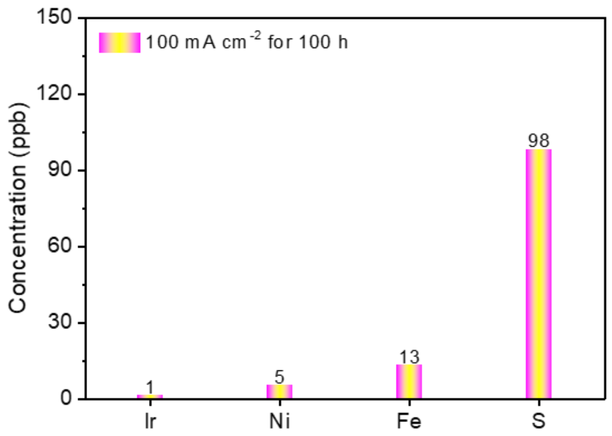

Supplementary Fig. 32 Quantify corrosion by means of *e.g.* inductively coupled mass spectrometry (ICP-MS). The concentration of different elements content in alkaline electrolytes after stability test at 100 mA cm^{-2} for 100 h.

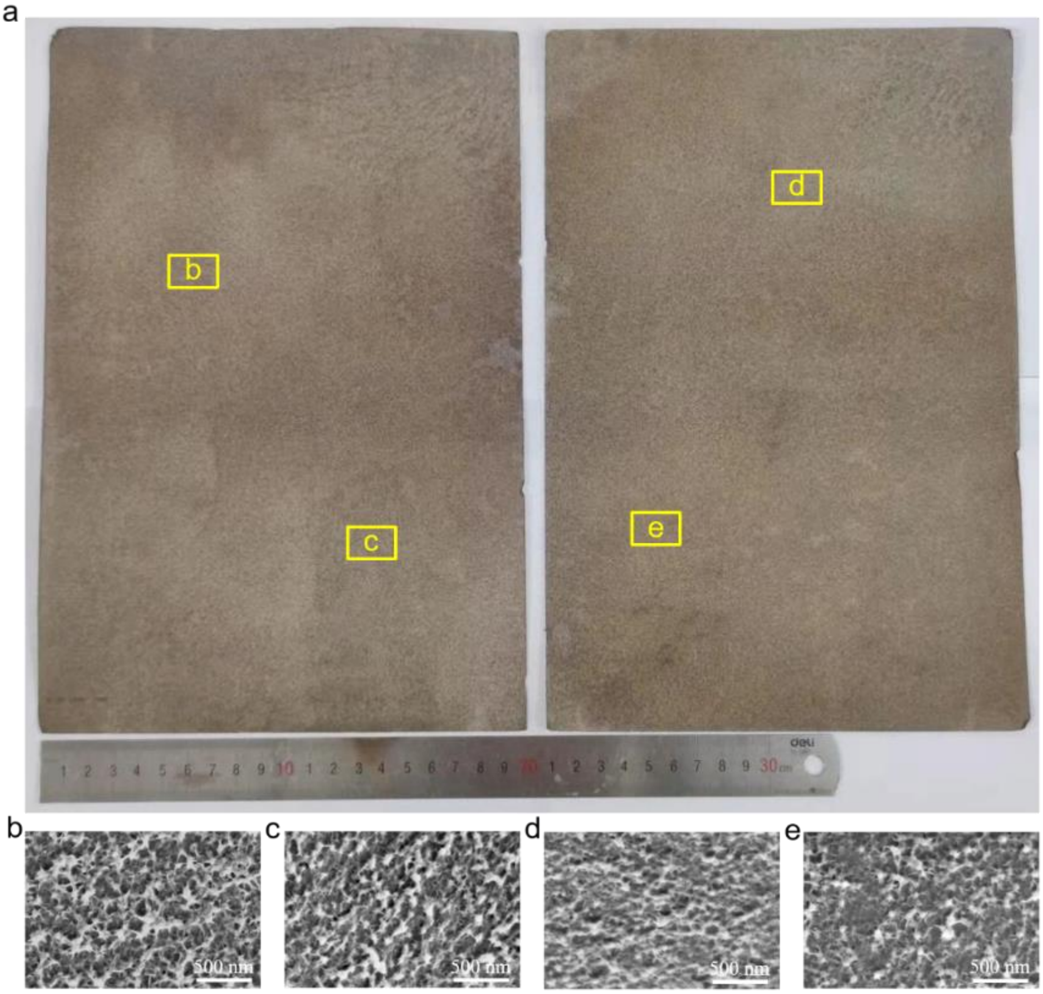

Supplementary Fig. 33 The morphology of the large-scale Ir_1/NFS electrode. a Digital image of Ir_1/NFS (20 cm × 30 cm) obtained from a scaled-up electrodeposition method. **b** - **e** The corresponding SEM images of the representative areas highlighted by yellow rectangles. Scale bar, **b** - **e** 500 nm.

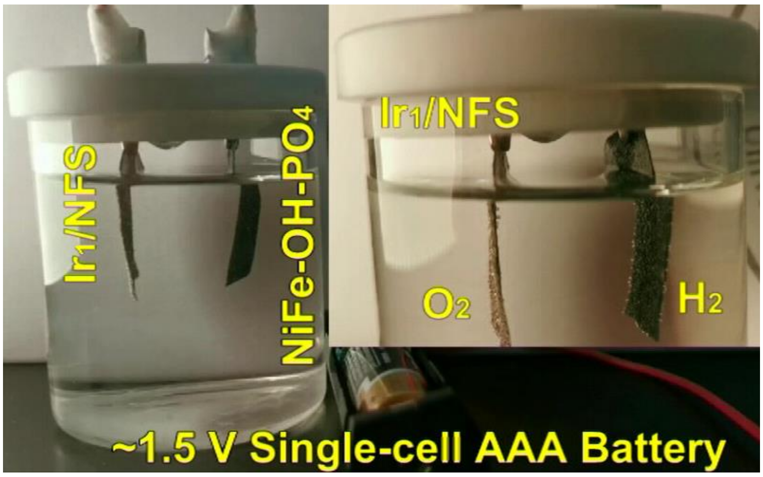

Supplementary Fig. 34 High-performance Ir_1/NFS anode for water splitting with a AAA battery. The optical photograph of alkaline water splitting at ~1.5 V shows the oxygen and hydrogen bubble generation by a AAA battery (Inset: close up of the electrodes). The electrodes of Ir_1/NFS and NiFe-OH-PO_4 area are 2 cm × 2 cm, respectively.

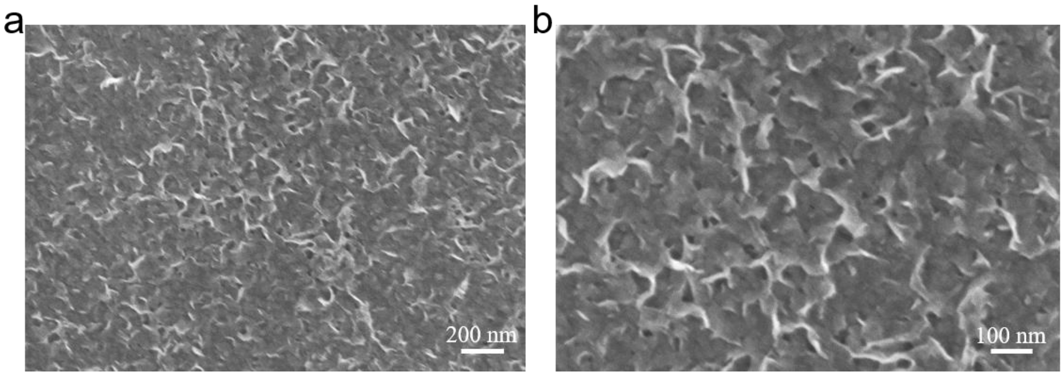

Supplementary Fig. 35 The morphology of the post-OER Ir_1/NFS. SEM images of the post-OER Ir_1/NFS. Scale bar, **a** 200 nm and **b** 100 nm.

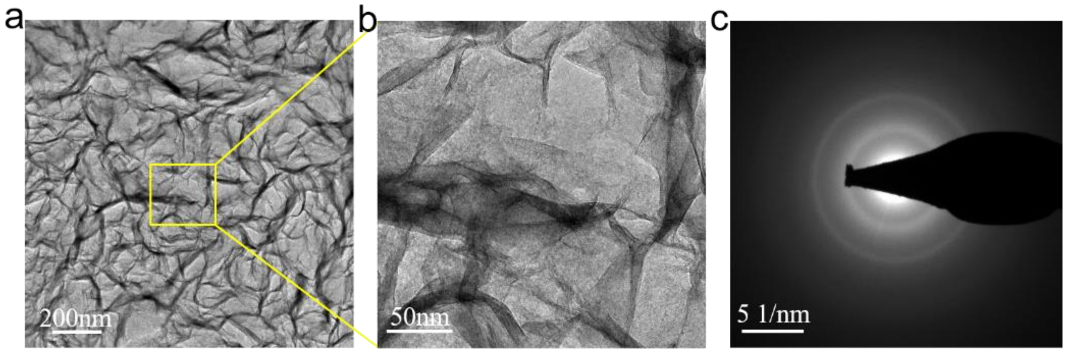

Supplementary Fig. 36 TEM images of the post-OER Ir_1/NFS. a and **b** TEM images of the post-OER Ir_1/NFS scratched off from the Ni foam electrode. **c** The corresponding selected area diffraction pattern. Scale bar, **a** 200 nm, **b** 50 nm, and **c** 5 1/nm.

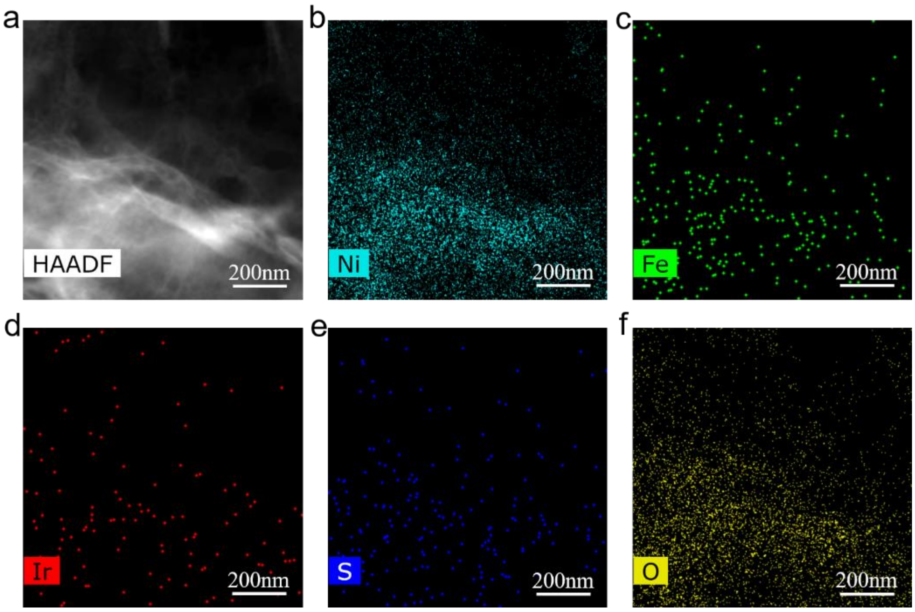

Supplementary Fig. 37 HAADF image and EDX elemental mappings of the post-OER Ir_1/NFS. **a** The HAADF-STEM images of the post-OER Ir_1/NFS and corresponding elemental distribution maps of **b** Ni, **c** Fe, **d** Ir, **e** S, and **f** O. Scale bar, **a** - **f** 200 nm.

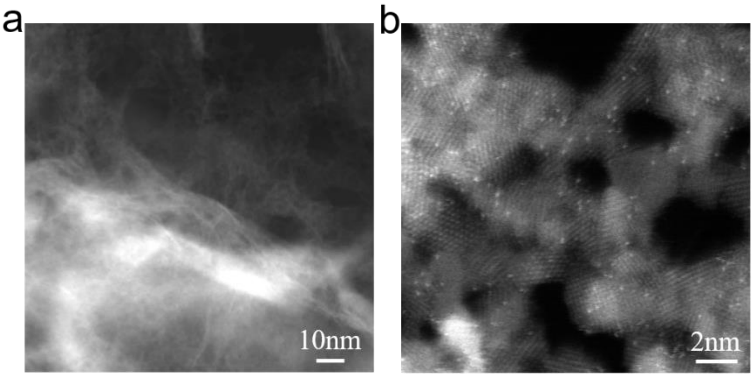

Supplementary Fig. 38 HAADF-STEM image of the post-OER Ir_1/NFS. **a** The HAADF-STEM image of the post-OER Ir_1/NFS. **b** The Cs-corrected STEM image of the post-OER Ir_1/NFS shows the monoatomic iridium dispersed on the surface of substrate (The bright dots corresponding to iridium single atoms are observed). Scale bar, **a** 10 nm and **b** 2 nm.

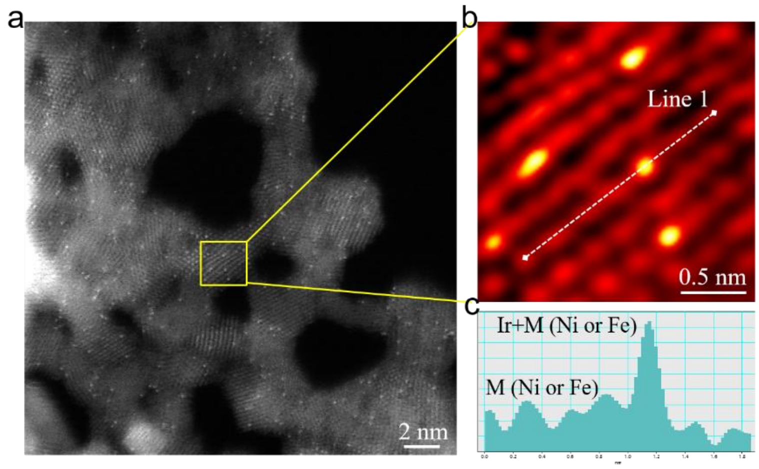

Supplementary Fig. 39 HAADF-STEM images of the post-OER Ir_1/NFS. a and **b** The HAADF-STEM images of the post-OER Ir_1/NFS. **c** Intensity line profiles taken along the corresponding white line 1 in **b**. Scale bar, **a** 2 nm and **b** 0.5 nm.

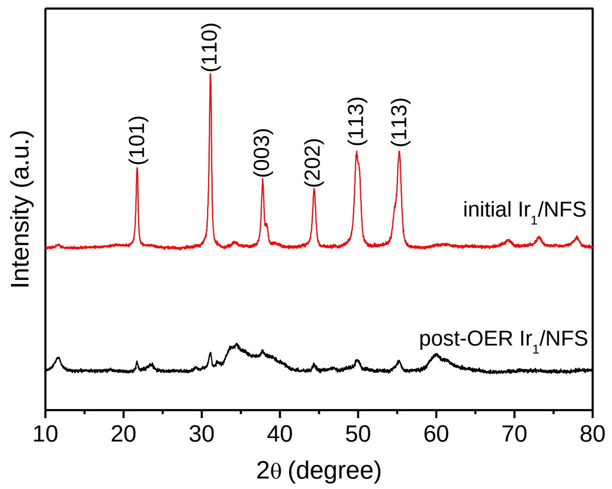

Supplementary Fig. 40 XRD characterizations. XRD patterns of the initial Ir_1/NFS and post-OER Ir_1/NFS. XRD patterns show that almost all the diffraction peaks of post-OER Ir1/NFS are weakened after the OER process at an overpotential of 250 mV for 10 h, indicating that the NFS substrate is transformed into an amorphous structure.

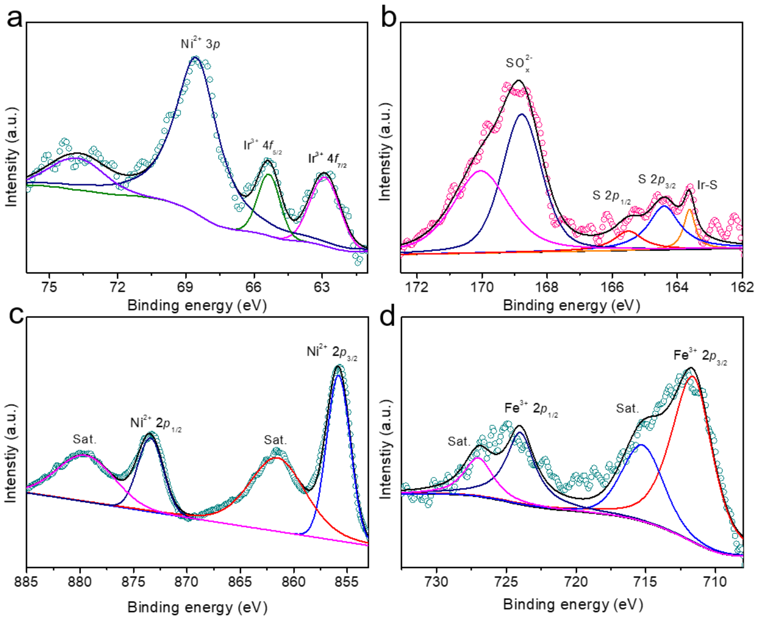

Supplementary Fig. 41 XPS characterizations. XPS spectra of **a** Ir 4*f*, **b** S 2*p*, **c** Ni 2*p*, and **d** Fe 2*p* regions with fitting curves for the post-OER Ir_1/NFS. The post-OER Ir_1/NFS shows the binding energy of Ir^{3+} $4f_{7/2}$ at 63.0 eV, which is slightly higher than the Ir_1/NFS of Ir^{3+} $4f_{7/2}$. It should be noted that the Ir-S-M (M stands for Ni or Fe) bond is still be observed in the post-OER Ir_1/NFS sample, indicating that the Ir-S-M bond is relatively stable during the OER process.

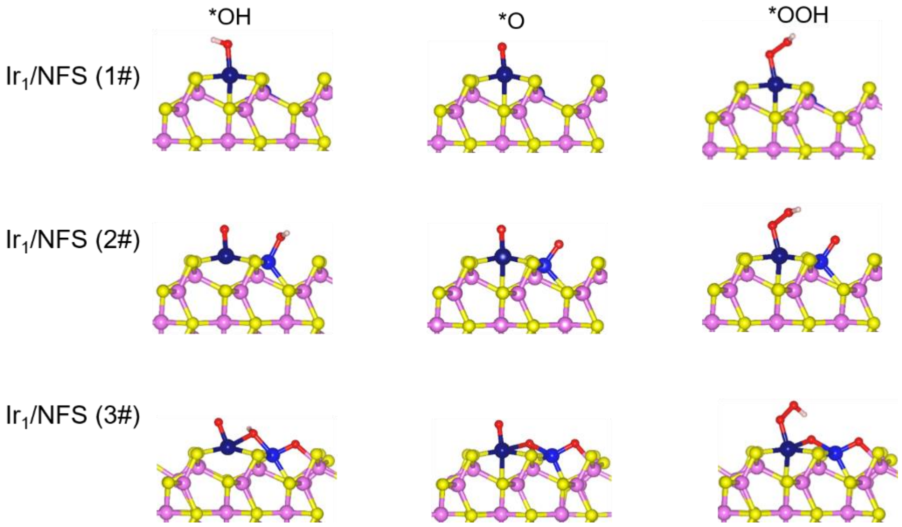

Supplementary Fig. 42 Calculation of the adsorption structures of different oxygen species on Ir_1/NFS. Three structural models corresponding to each specific oxygen species on Ir_1/NFS, respectively. In order to simulate the environment where oxygen is enriched on the electrode surface in actual electrocatalytic process, both Ir and Fe atoms have been highly coordinated by O atoms.

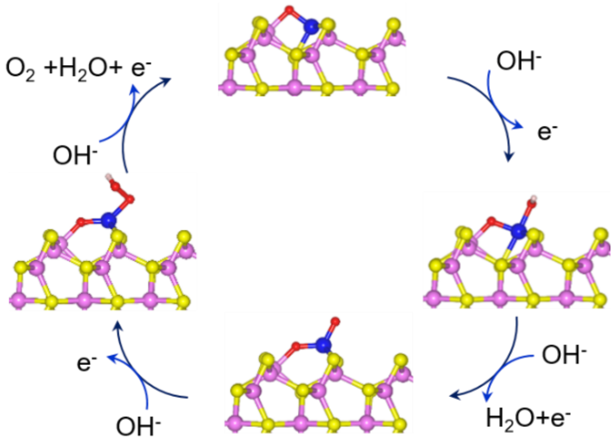

Supplementary Fig. 43 Caculated OER mechanism of NFS. Simulate the 4e^{-} mechanism of oxygen evolution reaction on NFS by the spin-polarized density functional theory

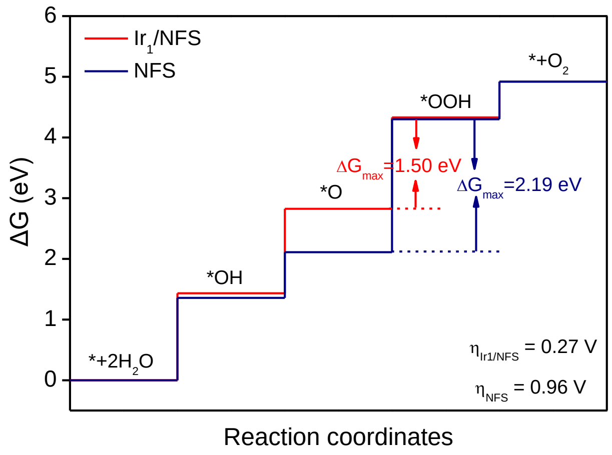

Supplementary Fig. 44 Corresponding Gibbs free energy change of Ir1/NFS and NFS. The free energy profiles of four-step elementary reaction on Ir1/NFS and NFS. The Fe site in the NFS on the plane surface showed a higher Gibbs free energy (2.19 eV) of the rate determining step than that of the Ir atom sites on the NFS (1.50 eV). Therefore, the Ir1/NFS possesses a better activity compared with that of NFS.

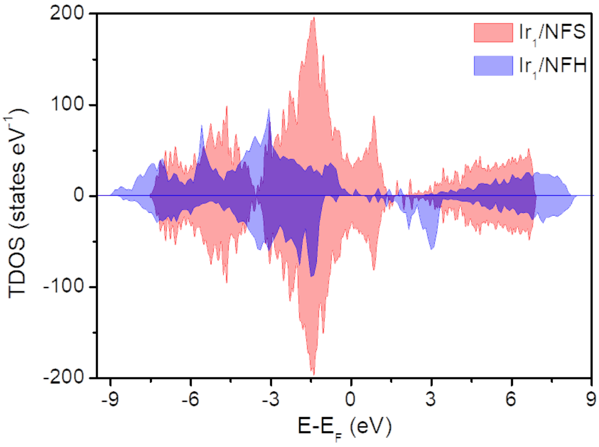

Supplementary Fig. 45 Calculated TDOS of Ir_1/NFS and Ir_1/NFH. The total charge density of states of Ir_1/NFS and Ir_1/NFH. The results display that Ir_1/NFS has more electronic states near the Fermi level compared to Ir_1/NFH, resulting in higher electrical conductivity.

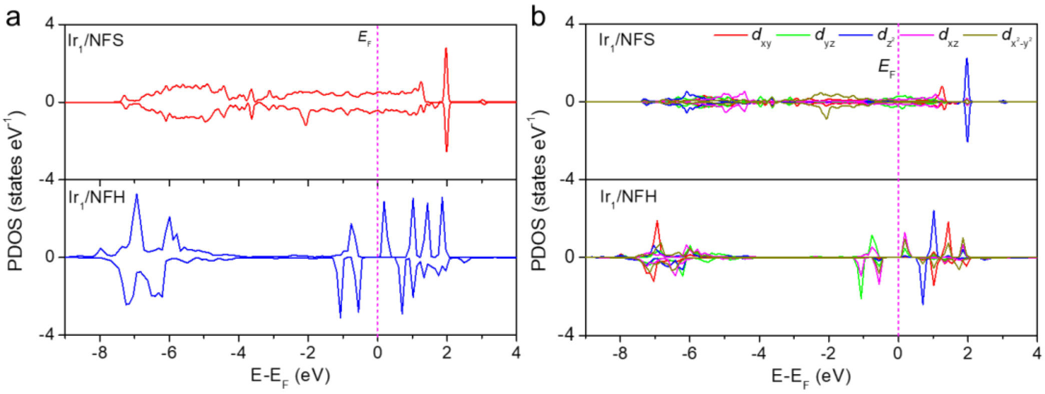

Supplementary Fig. 46 Calculated PDOS of Ir 4*d* orbital in Ir_1/NFS and Ir_1/NFH.

The PDOS results suggest that the electrons on Ir atoms in Ir_1/NFS are closer to the Fermi level, and tend to be delocalized throughout the reaction compared with Ir_1/NFH.

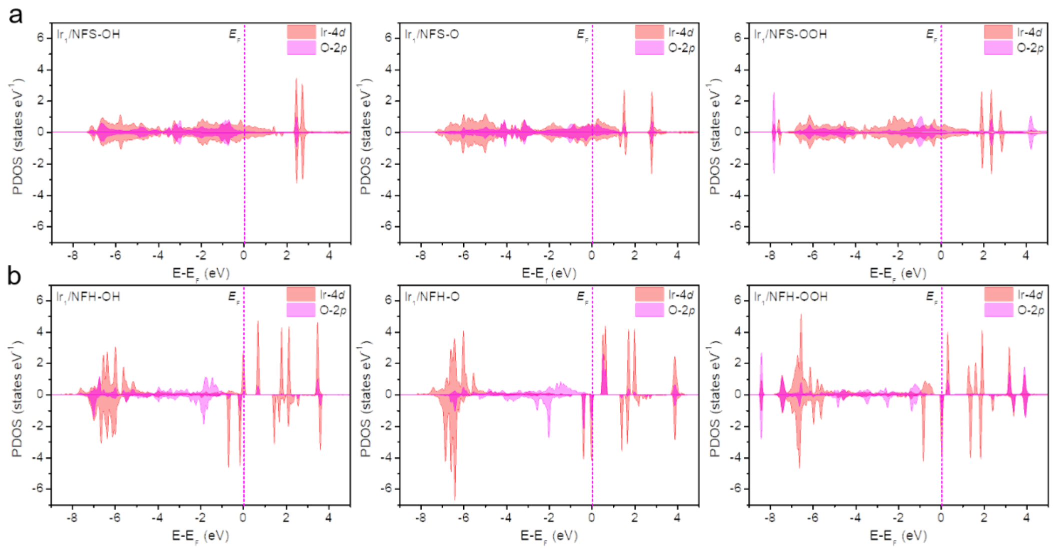

Supplementary Fig. 47 Calculated PDOS of Ir_1/NFS and Ir_1/NFH. PDOS for the 4*d* orbitals of Ir atoms and the 2*p* orbitals of the O atoms of the intermediate connected to it in the structure corresponding to three intermediates (*OH, *O, and *OOH) during the oxygen evolution reaction in **a** Ir_1/NFS and **b** Ir_1/NFH.

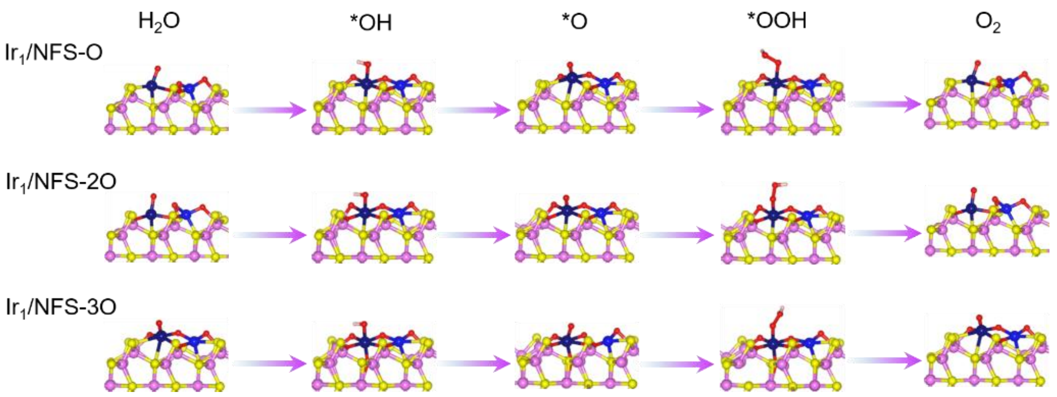

Supplementary Fig. 48 Adsorption structures of different oxygen species on Ir$_1$/NFS-nO (n=1, 2, 3). Proposed 4e$^-$ mechanism of oxygen evolution reaction on Ir$_1$/NFS-1O, Ir$_1$/NFS-2O, and Ir$_1$/NFS-3O. Three structural models were analyzed by replacing sulfur atoms near iridium atoms with oxygen atoms to simulate the influence of the oxidizing environment.

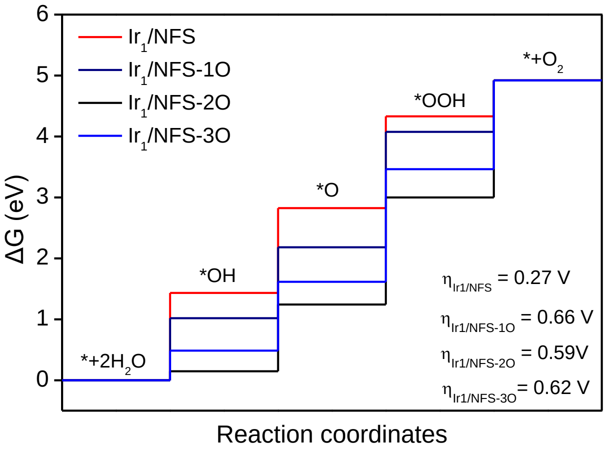

Supplementary Fig. 49 Corresponding Gibbs free energy change of Ir_1/NFS, and Ir_1/NFS-nO (n=1, 2, 3). The free energy profiles of four-step elementary reaction on Ir_1/NFS, Ir_1/NFS-1O, Ir_1/NFS-2O, and Ir_1/NFS-3O. The three structural models of catalytic pathways further indicated the OER reactions on Ir_1/NFS proceed with much lower energy barriers than that on the Ir_1/NFS-nO (n= 1, 2, 3). The theoretical overpotential increased to varying degrees according to the number of oxygen atoms replaced.

Supplementary Table 1 The electrocatalytic performance of as-prepared Ir_1/NFS and Ir_1/NFH at 10 mA cm^{-2} comparing with some state-of-the-art precious SACs.

Catalyst	Overpotential (mV)	Tafel slope (mV dec^{-1})	Stability	Reference
Ir_1/NFS	170	33	350 h@100 mA cm^{-2}	This work
Ir_1/NFH	190	35	198 h@100 mA cm^{-2}	This work
np-Ir/NiFeO	197	29.6	80 h@1.43 V	Ref. S1
Ir-NiO	215	38	10 h@10 mA cm^{-2}	Ref. S2
NiFe/$Ir_{0.03}$ NW@NSs	200	44.6	12 h@10 mA cm^{-2}	Ref. S3
Ir_1/$Co_{0.8}Fe_{0.2}Se_2$	230	-	-	Ref. S4
Ir_1@Co/NC	263	163	-	Ref. S5
Ir@Co	273	99	10 h@10 mA cm^{-2}	Ref. S6
NiVIr-LDH	180	38	400 h@200 mA cm^{-2}	Ref. S7
Ru/CoFe-LDHs	198	39	24 h@200 mA cm^{-2}	Ref. S8
Co_3O_4-Ru_1	249	104	100 h@100 mA cm^{-2}	Ref. S9
^{s}Au/NiFe LDH	237	36	20 h@100 mA cm^{-2}	Ref. S10
Rh SAC-CuO NAs/CF	197	71.7	-	Ref. S11

Supplementary Table 2 The feeding amount of Ni and Fe source in the electrodeposited electrolytes of the $Ir_1/Ni_yFe_{(6-y)}S_x$ (y = 0 ~ 6) catalysts.

Sample	$Ir_1/Ni_0Fe_6S_x$	$Ir_1/Ni_1Fe_5S_x$	$Ir_1/Ni_2Fe_4S_x$	$Ir_1/Ni_3Fe_3S_x$	$Ir_1/Ni_4Fe_2S_x$	$Ir_1/Ni_5Fe_1S_x$	$Ir_1/Ni_6Fe_0S_x$
Ni	0	1 mM	2 mM	3 mM	4 mM	5 mM	6 mM
Fe	6 mM	5 mM	4 mM	3 mM	2 mM	1 mM	0

Supplementary Table 3 The electrocatalytic performance of as-prepared Ir_1/NFS at 10 mA cm^{-2} comparing with some state-of-the-art catalysts in alkaline electrolyte.

Catalyst	Overpotential (mV)	Stability	Reference
Ir_1/NFS	170	350 h@100 mA cm^{-2}	This work
Ir_1/NFH	190	198 h@100 mA cm^{-2}	This work
$NiMoO_x$/NiMoS	186	25 h@500 mA cm^{-2}	Ref. S12
$CoMoS_x$/NF	345@100 mA cm^{-2}	48 h@500 mA cm^{-2}	Ref. S13
FeCoNi-HNTAs	184	80 h@1.796 V	Ref. S14
MoS_2/Ni_3S_2	218	10 h@10 mA cm^{-2}	Ref. S15
Mo-Ni_3S_2/Ni_xP_y/NF	238@50 mA cm^{-2}	24 h@160 mA cm^{-2}	Ref. S16
Ni-Fe PBA (V_{CN})	283	6 h@1000 mA cm^{-2}	Ref. S17
G-FeCoW oxyhydroxides	191	550 h@30 mA cm^{-2}	Ref. S18
CS-NiFeCu	180	20 h@20 mA cm^{-2}	Ref. S19
$Ni_3Fe_{0.5}V_{0.5}$	264@100 mA cm^{-2}	60 h@100 mA cm^{-2}	Ref. S20
NiFeMo	201	-	Ref. S21
NiFeW	202	-	Ref. S21

Supplementary Table 4 Comparison of mass activity of Ir_1/NFS and Ir_1/NFH on Ni foam electrodes with recently reported catalysts.

Catalyst	Electrolyte	Mass activity (A g^{-1})	Reference
Ir_1/NFS	1.0 M KOH	20.34@220 mV	This work
Ir_1/NFH	1.0 M KOH	13.10@220 mV	This work
np-Ir/NiFeO	1.0 M KOH	39.32@300mV	Ref. S1
IrO_2	1.0 M KOH	0.30@300mV	Ref. S1
^{s}Au/NiFe LDH	1.0 M KOH	0.0649@280mV	Ref. S10
γ-CoOOH	1.0 M KOH	66.6@300mV	Ref. S22
5.4 nm-$NiFeO_xH_y$	1.0 M KOH	~8@300mV	Ref. S23
4.3%-NiFe MOF	1.0 M KOH	2@300mV	Ref. S24
S|NiN_x-PC/EG	1.0 M KOH	0.9418@350 mV	Ref. S25

Supplementary Table 5 Comparison of TOF of Ir_1/NFS and Ir_1/NFH on Ni/Au_{disc} electrodes with recently reported catalysts.

Catalyst	Electrolyte	TOF (s^{-1})	Reference
Ir_1/NFS	1.0 M KOH	9.85@300 mV	This work
Ir_1/NFH	1.0 M KOH	1.15@300 mV	This work
^{s}Au/NiFe LDH	1.0 M KOH	0.11@280 mV	Ref. S10
5.4 nm $NiFeO_xH_y$	1.0 M KOH	6.2@300 mV	Ref. S23
S|NiN_x-PC/EG	1.0 M KOH	10.89@350 mV	Ref. S25
Ir/NiO_x	1.0 M KOH	1.3@300 mV	Ref. S26
Ni-NHGF	1.0 M KOH	0.72@300 mV	Ref. S27
w-$Ni(OH)_2$	1.0 M KOH	0.74@250 mV	Ref. S28

Supplementary Table 6 Fitting parameters of EIS of Ir_1/NFS and Ir_1/NFH at an overpotential of 250 mV on Ni/Au_{disc} electrodes.

Catalyst	R_s (Ω)	R_{ct} (Ω)	CPE	n
Ir_1/NFS	6.53	26.47	0.0019	0.94
Ir_1/NFH	5.84	83.11	0.0019	0.94

Supplementary Table 7 The Gibbs free energy (eV) change of four-step elementary reaction on Ir_1/NFS and Ir_1/NFH during OER process.

Catalyst	ΔG_1	ΔG_2	ΔG_3	ΔG_4
Ir_1/NFS	1.43	1.40	1.50	0.59
Ir_1/NFH	0.51	1.27	1.59	1.55

Supplementary Table 8 Charge transfer from substrate to oxygen containing species and Ir atom during the transition*OH to *O and *O to *OOH on Ir_1/NFS and Ir_1/NFH.

Catalyst	*OH→*O	*O→*OOH
Ir_1/NFS	0.24	−0.21
Ir_1/NFH	0.15	−0.31

Supplementary Table 9 Gibbs free energy of adsorption (eV) of *O, *OH, and *OOH on Ir_1/NFS and Ir_1/NFH during OER process.

Catalyst	*OH	*O	*OOH
Ir_1/NFS	1.43	2.83	4.33
Ir_1/NFH	0.51	1.78	3.37

Supplementary References

1 Jiang, K. et al. Dynamic active-site generation of atomic iridium stabilized on nanoporous metal phosphides for water oxidation. *Nat. Commun.* **11**, 2701 (2020).

2 Wang, Q. et al. Ultrahigh-loading of Ir single atoms on NiO matrix to dramatically enhance oxygen evolution reaction. *J. Am. Chem. Soc.* **142**, 7425-7433 (2020).

3 Luo, X. et al. Single-atom Ir-anchored 3D amorphous NiFe nanowire@nanosheets for boosted oxygen evolution reaction. *ACS Appl. Mater. Interfaces* **12**, 3539-3546 (2020).

4 Zhang, Z. R. et al. Electrochemical deposition as a universal route for fabricating single-atom catalysts. *Nat. Commun.* **11**, 1215 (2020).

5 Lai, W. H. et al. General π-electron-assisted strategy for Ir, Pt, Ru, Pd, Fe, Ni single-atom electrocatalysts with bifunctional active sites for highly efficient water splitting. *Angew. Chem. Int. Ed. Engl.* **58**, 11868-11873 (2019).

6 Babu, D. D. et al. Atomic iridium@cobalt nanosheets for dinuclear tandem water oxidation. *J. Mater. Chem. A* **7**, 8376-8383 (2019).

7 Wang, D. W. et al. Atomic and electronic modulation of self-supported nickel-vanadium layered double hydroxide to accelerate water splitting kinetics. *Nat. Commun.* **10**, 3899 (2019).

8 Li, P. S. et al. Boosting oxygen evolution of single-atomic ruthenium through electronic coupling with cobalt-iron layered double hydroxides. *Nat. Commun.* **10**, 1711 (2019).

9 Zhao, C. T. et al. Insights into the electronic origin of enhancing the catalytic activity

of Co_3O_4 for oxygen evolution by single atom ruthenium. *Nano Today* **34**, 100955 (2020).

10 Zhang, J. F. et al. Single-atom Au/NiFe layered double hydroxide electrocatalyst: probing the origin of activity for oxygen evolution reaction. *J. Am. Chem. Soc.* **140**, 3876-3879 (2018).

11 Xu, H. T. et al. Cation exchange strategy to single-atom noble-metal doped CuO nanowire arrays with ultralow overpotential for H_2O splitting. *Nano Lett.* **20**, 5482-5489 (2020).

12 Zhai, P. L. et al. Engineering active sites on hierarchical transition bimetal oxides/sulfides heterostructure array enabling robust overall water splitting. *Nat. Commun.* **11**, 5462 (2020).

13 Shan, X. Y. et al. An engineered superhydrophilic/superaerophobic electrocatalyst composed of the supported $CoMoS_x$ chalcogel for overall water splitting. *Angew. Chem. Int. Ed.* **59**, 1659-1665 (2020).

14 Li, H. Y. et al. Systematic design of superaerophobic nanotube-array electrode comprised of transition-metal sulfides for overall water splitting. *Nat. Commun.* **9**, 2452 (2018).

15 Zhang, J. et al. Interface engineering of MoS_2/Ni_3S_2 heterostructures for highly enhanced electrochemical overall-water-splitting activity. *Angew. Chem. Int. Ed.* **55**, 6702-6707 (2016).

16 Luo, X. et al. Interface engineering of hierarchical branched Mo-doped Ni_3S_2/Ni_xP_y hollow heterostructure nanorods for efficient overall water splitting. *Adv. Energy*

Mater. **10**, 1903891 (2020).

17 Yu, Z. Y. et al. Unconventional CN vacancies suppress iron-leaching in Prussian blue analogue pre-catalyst for boosted oxygen evolution catalysis. *Nat. Commun.* **10**, 2799 (2019).

18 Zhang, B. et al. Homogeneously dispersed multimetal oxygen-evolving catalysts. *Science* **352**, 333-337 (2016).

19 Zhang, P. L. et al. Dendritic core-shell nickel-iron-copper metal/metal oxide electrode for efficient electrocatalytic water oxidation. *Nat. Commun.* **9**, 381 (2018).

20 Jiang, J. et al. Atomic-level insight into super-efficient electrocatalytic oxygen evolution on iron and vanadium co-doped nickel (oxy)hydroxide. *Nat. Commun.* **9**, 2885 (2018).

21 Zhang, B. et al. High-valence metals improve oxygen evolution reaction performance by modulating 3d metal oxidation cycle energetics. *Nat. Catal.* **3**, 985-992 (2020).

22 Huang, J. H. et al. CoOOH nanosheets with high mass activity for water oxidation. *Angew. Chem. Int. Ed.* **54**, 8722-8727 (2015).

23 Roy, C. et al. Impact of nanoparticle size and lattice oxygen on water oxidation on $NiFeO_xH_y$. *Nat. Catal.* **1**, 820-829 (2018).

24 Cheng, W. R. et al. Lattice-strained metal–organic-framework arrays for bifunctional oxygen electrocatalysis. *Nat. Energy* **4**, 115-122 (2019).

25 Hou, Y. et al. Atomically dispersed nickel-nitrogen-sulfur species anchored on porous carbon nanosheets for efficient water oxidation. *Nat. Commun.* **10**, 1392

(2019).

26 Wang, Q. et al. Ultrahigh-loading of Ir single atoms on NiO matrix to dramatically enhance oxygen evolution reaction. *J. Am. Chem. Soc.* **142**, 7425-7433 (2020).

27 Fei, H. L. et al. General synthesis and definitive structural identification of MN_4C_4 single-atom catalysts with tunable electrocatalytic activities. *Nat. Catal.* **1**, 63-72 (2018).

28 Yan, J. Q. et al. Single atom tungsten doped ultrathin α-$Ni(OH)_2$ for enhanced electrocatalytic water oxidation. *Nat. Commun.* **10**, 2149 (2019).
