# Supplementary material for: Coordination modulation of iridium single-atom catalyst maximizing water oxidation activity
Source: Nat Commun. 2022 Jan 10;13:24. doi: 10.1038/s41467-021-27664-z (PMC8748886; doi:10.1038/s41467-021-27664-z)
Supplement: Supplementary file 2 — Description of Additional Supplementary Files [file 41467_2021_27664_MOESM2_ESM.docx]

Description of Additional Supplementary Files

File name: Supplementary Movie 1. The movie of water splitting by 1.5 V single-cell AAA battery.

Description: The large-area Ir1/NFS and NiFe-OH-PO4 electrodes (2 cm  2 cm) were used as oxygen and hydrogen evolution catalysts, which were used to build up a two-electrode electrolysis device for water electrolysis. The demonstrate the overall electrochemical water splitting could be driven by a single-cell AAA battery with a nominal voltage of ~1.5 V.
